# Frequency down-conversion based on optical cascading process—New effective way for generation of far infrared or THz radiation

**DOI:** 10.1371/journal.pone.0268228

**Published:** 2022-10-14

**Authors:** Vyacheslav A. Trofimov, Dmitry M. Kharitonov, Mikhail V. Fedotov

**Affiliations:** 1 South China University of Technology, Guangzhou, China; 2 Lomonosov Moscow State University, Leninskye Gory, Moscow, Russia; Rutgers University Newark, UNITED STATES

## Abstract

Infrared and THz optics has many promising practical applications such as in spectroscopy, diagnostic, optical metrology, sensing, and many others. Due to limited number of IR radiation sources, the frequency down-conversion processes are widely used for obtaining infrared radiation. Among them, the most applicable method is a generation of wave with difference frequency under the three-waves interaction in a medium with quadratic nonlinear response. Below we propose a new effective tool for three times decreasing frequency of the incident pulse based on three-waves interaction in a medium with the quadratic susceptibility. At such interaction, a medium’s response inherent cubic non-linearity appears due to so-called cascading SHG. The frequency down-conversion process possesses two stable modes. This is shown using multi-scale method. For each of the modes, the analytical solution is developed in the framework of the long pulse duration approximation without using the pump energy non-depletion approximation. The computer simulation results confirm those of analytical analysis. We show that the conversion efficiency of the incidentpump pulse energy achieves about 70%, if the low frequency wave incident intensity equals zero, or almost 100%, if the incident intensity of the low frequency wave is non-zero. The developed theoretical approach may be applied to other processes of the frequency down-conversion.

## 1 Introduction

The problem of laser radiation generation in the infrared (IR) and mid-infrared (M-IR) range of the frequencies remains interesting for many researches. As is well-known, there are several approaches for getting IR radiation. The first of them is M-IR lasers. A full review, dedicated to M-IR lasers based on metal doped chalcogenides, can be found in [1]. The laser generates a radiation with the wavelengths up to 5.1 *μ*m.

Another approach consists in the frequency conversion due to non-linear response of a medium. The most commonly used process is a difference frequency generation (DFG) in a medium with quadratic susceptibility. In this case, the wave with difference frequency *ω*_1_ (often called as idler wave) is generated from the waves with frequencies *ω*_3_ (pump wave) and *ω*_2_ (signal wave):

$$\omega_{1}=\omega_{3}-\omega_{2},\;\left(\omega_{3}>\omega_{2}\right).$$

The equations, which describe this process, as well as their derivation from the Maxwell’s equations, can be found in [2], for example. There are also many papers, describing various methods of M-IR radiation generation, based on DFG process in various nonlinear crystals. So, a general description of the AgGaGe_5_Se_12_ crystal properties as well as M-IR radiation generation with the carrier frequency, equal difference between the frequencies of two other waves is presented in [3]. The energy of 5.1*μ*J at the wavelength λ_1_ = 5*μ*m was obtained at interacting pulses with the 50 *μ*J pump wave energy and 28 *μ*J energy of the signal wave. The computer simulations shows the possibility of 25% conversion efficiency. A frequency down-converter based on using two nonlinear crystals (AgGaS_2_ and AgGaSe_2_) is proposed in [4]. The MgO:LiNbO_3_ crystal is used in [5] to obtain femtosecond pulse tunable in the range 3.2−4.8*μm* of frequencies with maximal average power of 1.1*mW* using nonlinear mixing of the pulse 170*mW*, 65*fs* at a fixed wavelength of 1.58*μm* (pump) with the pulse 11.5*mW*, 40*fs* tunable in the near-infrared range of frequencies: 1.05 and 1.18*μm*. In [6], the DFG in GaAs crystal was realized, and as a result, a tunable source of M-IR CW radiation was obtained with maximal power of 51 mW on the frequency λ_1_ = 6543 nm. The input power of the pulses was 40 mW at the frequency λ_3_ = 2010 nm and at the frequency λ_2_ = 2900 nm. The experimental setup containing orientation patterned gallium phosphide crystal is presented in [7]. The M-IR radiation source with the wavelength tunable in the range 6–9 *μ*m is obtained with maximal average power of 7.4 mW on the frequency λ_1_ = 7.5 *μ*m under interaction of the pump pulse with wavelength 1570 nm and 175 mW of the average power and the signal pulse with the wavelength belonging to 1953–1965 nm range of the frequencies, and with the average power 152–235 mW.

Optical parametric oscillator (OPO) may be promising way for the IR radiation generation. In [8], the conversion efficiency 34% is achieved as the average output power of 7.7 W from the incident pump power of 23 W. Rotated Image Singly-Resonant Twisted RectAngle OPO setup is applied to obtain M-IR radiation pulse with the wavelength 6450 nm at using pump pulse with the wavelength 2 *μ*m. The conversion efficiency of this generation scheme is achieved up to 13%. The full review of DFG in the non-oxide crystals is presented in [9] along with many other references to the different works in this area.

Another possible way to obtain the IR radiation is the use of degenerate four wave mixing (DFWM) in a media with cubic non-linear response. In this case, the relation between frequencies of the interacting waves is the following:

$$\omega_{1}+\omega_{2}=2\omega_{3}.$$

There are various investigations in this direction during last two decades. For example, 1.4 m fused-silica photonic crystal was used in [10] to obtain the average power 450 mW on the frequency λ_1_ = 2539 nm, which is about 6% of the incident pump pulse average power. In [11] the authors obtained the efficiency 0.2% for the conversion of radiation with the wavelength λ_3_ = 1.064 *μ*m to one with the wavelength λ_1_ = 3.105*μ*m at the nanosecond pulse propagation in photonic crystal fiber. In [12], more, than 2% of the incident pump pulse energy at the frequency λ_3_ = 1.064*μ*m is converted to the idler pulse possessing the wavelength λ_1_ = 2929 nm in an endlessly single-mode silica fiber for the picosecond pulse. The problem of the frequency down-conversion is investigated also theoretically. In [13], the terahertz-wave generation in silicon membranes is discussed for the pump pulse wavelength λ_3_ = 4.3 *μ*m. It was predicted that the conversion efficiency of the pump energy to the idler wave with wavelength of 32.5 *μ*m (frequency equals 9.8684 THz) is 1.39%. Computer simulation for mid-infrared fiber optical parametric oscillators based on DFWM is provided in [14]. The authors showed a possibility of the conversion efficiency being greater than 10%.

Thus, we see that the frequency down-conversion is actual and this conversion requires increasing its efficiency. Current study is devoted to a special case of DFG, at which the pump wave has a tripled frequency with respect to the generated wave. Therefore, commonly used notations for the interacting waves are not convenient in our opinion. That is why we call the generated wave (we denote its frequency by *ω*_1_ = *ω*) as low frequency wave (LFW) and the pump wave, whose carrier frequency is *ω*_3_ = 3*ω*, as high frequency wave (HFW), and the signal wave, whose frequency equals *ω*_2_ = 2*ω*, as intermediate frequency wave (IFW).

The HFW falls on a medium with the quadratic susceptibility and propagates under the condition of the phase matching between HFW and LFW. This can be achieved in crystals, which are used for IR wave generation. For example, the computation, based on the results of paper [15], shows that this condition can be reached for LFW with the wavelength up to 10.2 *μ*m in AgGaS_2_ crystal. In the same time, phase mismatching between LFW and IFW (as well as between HFW and IFW) is relatively large. This allows us to realize in a medium, possessing the quadratic susceptibility, the response inherent the cubic non-linearity due to cascading process of the waves interaction. In our opinion, the cascading process is very promising tool for the frequency down-conversion.

We use multi-scale method for deriving the set of modified equations, which approximates the original problems, and demonstrate a possibility of the frequency down-conversion using proposed method. We find out an evolution of the intensities along their propagation coordinate and show that there is high-effective mode of the frequency down-conversion: almost 90% of the HFW energy can be converted to the LFW. Our analysis shows also that there are two modes of the LFW generation (or amplification) in dependence on the waves incident intensities. Moreover, at certain incident intensity of the IFW, the high-effective mode of the LFW generation occurs even if the incident LFW intensity equals zero-value.

The paper is organized as follows. We state the mathematical model describing the frequency down-conversion of the fundamental wave (HFW) to a generation of wave (LFW) with the frequency equal to one third of main frequency in a medium with quadratic nonlinear response. Then we apply multi-scale method and derive a set of the modified equations in the framework of big phase mismatching between IFW and other two waves. We investigate theoretically and on the base of computer simulation two cases: the IFW incident intensity equals zero or not. In both cases, we demonstrate a possibility achieving high efficient LFW amplification.

## 2 Problem statement

An interaction of three optical pulses with carrier frequencies *ω*, 2*ω*, 3*ω* in a medium with quadratic nonlinear response is described by the set of non-linear Schrödinger equations:

$$\begin{array}{c} \begin{array}{cc} & \frac{\partial A_{1}}{\partial z}+iD_{1}\frac{\partial^{2}A_{1}}{\partial t^{2}}+i(\gamma_{12}A_{1}^{*}A_{2}e^{-i\Delta_{21}kz}+\gamma_{23}A_{2}^{*}A_{3}e^{-i(\Delta_{31}k-\Delta_{21}k)z})=0,\\ & \frac{\partial A_{2}}{\partial z}+\nu_{21}\frac{\partial A_{2}}{\partial t}+iD_{2}\frac{\partial^{2}A_{2}}{\partial t^{2}}+i(\gamma_{11}A_{1}^{2}e^{i\Delta_{21}kz}+2\gamma_{13}A_{1}^{*}A_{3}e^{-i(\Delta_{31}k-\Delta_{21}k)z})=0,\\ & \frac{\partial A_{3}}{\partial z}+\nu_{31}\frac{\partial A_{3}}{\partial t}+iD_{3}\frac{\partial^{2}A_{3}}{\partial t^{2}}+3i\gamma_{21}A_{1}A_{2}e^{i(\Delta_{31}k-\Delta_{21}k)z}=0,\;0<z\leq L_{z},0<t<L_{t} \end{array} \end{array}$$ (1)

with the following initial condition and boundary conditions (BCs):

$$\begin{array}{c} \begin{array}{cc} & A_{1}\left(0,t\right)=A_{10}\left(t\right),\;A_{2}\left(0,t\right)=A_{20}\left(t\right),\;A_{3}\left(0,t\right)=A_{30}\left(t\right),\;t\in \left[0,L_{t}\right],\\ & A_{1}\left(z,0\right)=A_{2}\left(z,0\right)=A_{3}\left(z,0\right)=A_{1}\left(z,L_{t}\right)=A_{2}\left(z,L_{t}\right)=A_{3}\left(z,L_{t}\right)=0,\;z\in \left[0,L_{z}\right]. \end{array} \end{array}$$ (2)

Here *A*_1_, *A*_2_, *A*_3_ are the complex amplitudes of the LFW, IFW or HFW, respectively. Coefficients *γ*_*jl*_ characterize the nonlinear coupling of the interacting pulses at the corresponding frequencies. For simplicity, we neglect a difference between the coefficients:

$$\gamma_{jl}=\gamma ,\;j=1,\;2,\;l=1,\;2,\;3.$$

However, it does not restrict our analysis because all estimations can be provided in more general case. Parameter Δ_21_ *k* and Δ_31_ *k* characterize the phase mismatching between the IFW, HFW and LFW, respectively. Parameters *D*_*j*_, *j* = 1, 2, 3 and *ν*_*j*1_, *j* = 2, 3 are the dimensionless group-velocity dispersion (GVD) and group-velocity mismatching (GVM), respectively. Variable *z* is a dimensionless spatial coordinate along which the pulse propagates. *L*_*z*_ characterizes the pulse propagation distance. Variable *t* is a dimensionless time coordinate changing between 0 and *L*_*t*_.

The dimensionless parameters are expressed through the physical ones in the following way:

$$\begin{array}{c} \begin{array}{cc} & D_{j}=-\frac{1}{2}\frac{\partial^{2}\bar{k}}{\partial {\bar{\omega }}^{2}}{|}_{{\bar{\omega }}_{j}}\frac{Z_{n}}{\tau_{p}^{2}},\;A_{j}=\frac{{\bar{A}}_{J}}{A_{0}},\;j=1,2,3,\;\gamma_{jl}=\frac{2\pi \chi^{\left(2\right)}\left({\bar{\omega }}_{j},\;{\bar{\omega }}_{l}\right)\bar{k}A_{01}}{n^{2}\left({\bar{\omega }}_{j}\right)}Z_{n},\;j=1,2,\;l=1,\;2,\;3,\\ & \Delta_{21}k=\Delta_{21}\bar{k}Z_{n},\;\Delta_{31}k=\Delta_{31}\bar{k}Z_{n}. \end{array} \end{array}$$ (3)

where *τ*_*p*_ is the incident pulse duration at the low frequency $\bar{\omega }$; *Z*_*n*_ is a normalization length chosen to be equal 4 mm, $\chi^{\left(2\right)}\left({\bar{\omega }}_{j},{\bar{\omega }}_{l}\right))$ is the quadratic susceptibility of a medium at an interaction of waves with the frequencies $\bar{\omega_{j}},\;{\bar{\omega }}_{l}$. Parameter $\bar{k}$ is a dimensional wave-number of the LFW. $\Delta_{21}\bar{k}$ and $\Delta_{31}\bar{k}$ are dimensional phase mismatching between the IFW or the HFW and the LFW, respectively. *A*_0_ is a normalization value, which will be precised further. ${\bar{A}}_{j}$ are the envelope of the wave packets measured in physical units.

Because in this paper the frequency down-conversion is of interest, then we suppose occurring phase matching between the LFW and HFW:

$$\Delta_{31}k=0.$$

In turn, the large phase mismatching between the LFW and IFW occurs. Under such condition the multi-scale method is very effective for the frequency conversion process analysis and we use this method for deriving approximate equations.

## 3 Set of modified equations

Under large phase mismatching between LFW and IFW (Δ_21_ *k*), the solution of the problem (1), (2) can be approximated in the following way:

$$\begin{array}{c} \begin{array}{cc} & A_{1}=U+\frac{1}{\Delta_{21}k}(\gamma \left(U^{*}Ve^{-i\Delta_{21}kz}-V^{*}We^{i\Delta_{21}kz}\right)+u_{1}),\\ & A_{2}=V+\frac{1}{\Delta_{21}k}(-\gamma \left(U^{2}+2U^{*}W\right)e^{i\Delta_{21}kz}+v_{1}),\\ & A_{3}=W+\frac{1}{\Delta_{21}k}(3\gamma UVe^{-i\Delta_{21}kz}+w_{1}), \end{array} \end{array}$$ (4)

with accuracy of *O*((Δ_21_ *k*)^−2^) by using multi-scale method. Here, the functions *U*, *V*, *W* are governed by the following set of equations:

$$\begin{array}{c} \begin{array}{cc} & \frac{\partial U}{\partial z}+iD_{1}\frac{\partial^{2}U}{\partial t^{2}}-i\tilde{\alpha }{\left(|U\right|}^{2}U+3U^{*2}W-{4U|V|}^{2}+{2U|W|}^{2})=0,\\ & \frac{\partial V}{\partial z}+\nu_{21}\frac{\partial V}{\partial t}+iD_{2}\frac{\partial^{2}V}{\partial t^{2}}+2i\tilde{\alpha }{\left(4|U\right|}^{2}-{\left|W\right|}^{2})V=0,\\ & \frac{\partial W}{\partial z}+\nu_{31}\frac{\partial W}{\partial t}+iD_{3}\frac{\partial^{2}W}{\partial t^{2}}-3i\tilde{\alpha }(U^{3}+{2|U|}^{2}W+{\left|V\right|}^{2}W)=0. \end{array} \end{array}$$ (5)

Parameter $\tilde{\alpha }$ is expressed through the non-linear coupling coefficient *γ* and the phase mismatching Δ_21_ *k* as $\tilde{\alpha }=\frac{\gamma^{2}}{\Delta_{21}k}$. The functions *u*_1_, *v*_1_, *w*_1_ are the linear equations solutions:

$$\begin{array}{c} \begin{array}{cc} & \frac{\partial u_{1}}{\partial z}+iD_{1}\frac{\partial^{2}u_{1}}{\partial t^{2}}=0,\\ & \frac{\partial v_{1}}{\partial z}+\nu_{21}\frac{\partial v_{1}}{\partial t}+iD_{2}\frac{\partial^{2}v_{1}}{\partial t^{2}}=0,\\ & \frac{\partial w_{1}}{\partial z}+\nu_{31}\frac{\partial w_{1}}{\partial t}+iD_{3}\frac{\partial^{2}w_{1}}{\partial t^{2}}=0. \end{array} \end{array}$$ (6)

The derivation of these equations is presented in Appendix A and is made in a manner similar to [16]. The initial conditions and BCs for the functions introduced above are written as follows:

$$\begin{array}{c} \begin{array}{cc} & U\left(0,t\right)=A_{10}\left(t\right),\;V\left(0,t\right)=A_{20}\left(t\right),\;W\left(0,t\right)=A_{30}\left(t\right),\\ & u_{1}\left(0,t\right)=\gamma (A_{20}^{*}\left(t\right)A_{30}\left(t\right)-A_{10}^{*}\left(t\right)A_{20}\left(t\right)),\;v_{1}\left(0,t\right)=\gamma (A_{10}^{2}\left(t\right)+2A_{10}^{*}\left(t\right)A_{30}\left(t\right)),\\ & w_{1}\left(0,t\right)=-3\gamma A_{10}\left(t\right)A_{20}\left(t\right),\;t\in \left[0,L_{t}\right],\\ & U\left(z,0\right)=V\left(z,0\right)=W\left(z,0\right)=U\left(z,L_{t}\right)=V\left(z,L_{t}\right)=W\left(z,L_{t}\right)=0,\\ & u_{1}\left(z,0\right)=v_{1}\left(z,0\right)=w_{1}\left(z,0\right)=u_{1}\left(z,L_{t}\right)=v_{1}\left(z,L_{t}\right)=w_{1}\left(z,L_{t}\right)=0,\;z\in \left[0,L_{z}\right]. \end{array} \end{array}$$ (7)

The set of Eq (5) possesses some conservation laws (invariants or integrals of motion):

$$\begin{array}{c} \begin{array}{cc} & I_{1UW}=\int_{0}^{L_{t}}({\left|U\right|}^{2}+{\left|W\right|}^{2})dt=const,\\ & I_{1V}=\int_{0}^{L_{t}}{\left|V\right|}^{2}dt=const,\\ & I_{3}=\int_{0}^{L_{t}}(3\nu_{21}Im(V^{*}\frac{\partial V}{\partial t})+2\nu_{31}Im(W^{*}\frac{\partial W}{\partial t})-6D_{1}{\left|\frac{\partial U}{\partial t}\right|}^{2}-3D_{2}{\left|\frac{\partial V}{\partial t}\right|}^{2}-2D_{3}{\left|\frac{\partial W}{\partial t}\right|}^{2}-\\ - & 3\tilde{\alpha }(4Re\left(U^{3}W^{*}\right)+{\left|U\right|}^{4}+{4|U|}^{2}{\left|W\right|}^{2}-{8|U|}^{2}{\left|V\right|}^{2}+{2|V|}^{2}{\left|W\right|}^{2}))dt=const. \end{array} \end{array}$$ (8)

The first two invariants characterize the energy of pulses. The third invariant is Hamiltonian of the waves interaction. These conservation laws will be used for developing analytical solution of the problem (5) in the framework of long pulse duration approximation. It should be stressed that the set of Eq (1) also possesses some conservation laws.

## 4 Long pulse duration approximation

In this section we derive the analytical solution of the problem (5) neglecting temporal derivatives. In this case, all functions depend only on the longitudinal coordinate *z*:

U=U(z),V=V(z),W=W(z).

Therefore, the problem (5), (7) can be rewritten as follows:

$$\begin{array}{c} \begin{array}{cc} & \frac{dU}{dz}-i\tilde{\alpha }{\left(|U\right|}^{2}U+3U^{*2}W-{4U|V|}^{2}+{2U|W|}^{2})=0,\\ & \frac{dV}{dz}+2i\tilde{\alpha }{\left(4|U\right|}^{2}-{\left|W\right|}^{2})V=0,\\ & \frac{dW}{dz}-3i\tilde{\alpha }(U^{3}+{2|U|}^{2}W+{\left|V\right|}^{2}W)=0,\\ & U\left(0\right)=A_{10},\;V\left(0\right)=A_{20},\;W\left(0\right)=A_{30}. \end{array} \end{array}$$ (9)

The conservation laws (8) transform to the kind:

$$\begin{array}{c} \begin{array}{cc} & I_{1UW}={\left|U\right|}^{2}+{\left|W\right|}^{2}=1,\\ & I_{1V}={\left|V\right|}^{2}={\left|A_{20}\right|}^{2},\\ & I_{3}=-3\tilde{\alpha }(4Re\left(U^{3}W^{*}\right)+{\left|U\right|}^{4}+{4|U|}^{2}{\left|W\right|}^{2}-{8|U|}^{2}{\left|V\right|}^{2}+{2|V|}^{2}{\left|W\right|}^{2})=const. \end{array} \end{array}$$ (10)

Let us do some notes about the invariants. We choose value of the first invariant to be equal to unity. It means that the normalization value *A*_0_ in (3) is chosen equal to squared root from sum of the HFW and LFW maximal intensities. It should be also stressed that the IFW intensity remains unchanged at its propagation (see (10)). Despite this, the IFW intensity influences significantly the frequency down-conversion process as it will be shown below.

To solve the Eq (9), let us represent the complex amplitudes in following way:

$$\begin{array}{c} U\left(z\right)=a_{1}\left(z\right)\text{exp}\left(i\varphi_{1}\left(z\right)\right),\;V\left(z\right)=a_{20}\text{exp}\left(i\varphi_{2}\left(z\right)\right),\;W\left(z\right)=a_{3}\left(z\right)\text{exp}\left(i\varphi_{3}\left(z\right)\right), \end{array}$$ (11)

where *a*_1_, *a*_3_, *φ*_*j*_, *j* = 1, 2, 3 are real-valued function and *a*_20_ = |*A*_20_|. Thus, the problem (9) takes the form

$$\begin{array}{c} \begin{array}{cc} & \frac{da_{1}}{dz}=-3\tilde{\alpha }a_{1}^{2}a_{3}\text{sin}\varphi ,\\ & \frac{da_{3}}{dz}=3\tilde{\alpha }a_{1}^{3}\text{sin}\varphi ,\\ & \frac{d\varphi }{dz}-\tilde{\alpha }(3(\frac{a_{1}^{3}}{a_{3}}-3a_{1}a_{3})\text{cos}\varphi +3a_{1}^{2}-6a_{3}^{2}+15a_{20}^{2})=0,\\ & \frac{d\varphi_{2}}{dz}+2i\tilde{\alpha }\left(4a_{1}^{2}-a_{3}^{2}\right)=0,\\ & a_{1}\left(0\right)=|A_{10}|,\;a_{3}\left(0\right)=\left|A_{30}\right|,\;\varphi \left(0\right)=\varphi_{0},\;\varphi_{2}\left(0\right)=\text{arg}\left(A_{20}\right). \end{array} \end{array}$$ (12)

Here *φ* = *φ*_3_−3*φ*_1_ is a phase difference between the HFW and LFW, *φ*_0_ is its value in the input section of a medium. As one can see, *φ*_2_ does not influence the LFW amplitude *a*_1_, so we do not take it into account below. On the other hand, the third Eq (12) contains a term with the IFW amplitude *a*_20_ and, therefore, the phase difference *φ* depends on it also. Consequently, the IFW intensity influences the LFW intensity.

The invariants (10) are transformed to a form:

$$\begin{array}{cc} & I_{1_{a_{1}a_{3}}}=a_{1}^{2}+a_{3}^{2}=1,\\ & I_{1_{a_{2}}}=a_{2}^{2}=a_{20}^{2},\\ & I_{3}=3\tilde{\alpha }(-4a_{1}^{3}a_{3}\text{cos}\varphi -a_{1}^{4}-4a_{1}^{2}a_{3}^{2}+8a_{20}{}^{2}a_{1}^{2}-2a_{20}^{2}a_{3}^{2}). \end{array}$$

Let us stress that we took into account the invariant $I_{1_{a_{2}}}$ at writing representation (11). Further we modify the Hamiltonian by dividing it on $3\tilde{\alpha }$:

$$\begin{array}{c} {\tilde{I}}_{3}=-4a_{1}^{3}a_{3}\text{cos}\varphi -a_{1}^{4}-4a_{1}^{2}a_{3}^{2}+8a_{20}{}^{2}a_{1}^{2}-2a_{20}^{2}a_{3}^{2}={\tilde{I}}_{30} \end{array}$$ (13)

Then, using this relation, the phase difference can be expressed through the pulses amplitudes. Thus, it is possible to integrate the ordinary differential equation with respect to the LFW intensity.

Firstly, we consider a special case *a*_20_ = 0 to identify the main features of the frequency down-conversion process through cascading second harmonic generation (SHG).

### 4.1 IFW intensity is absent (*a*_20_ = 0)

#### 4.1.1 Analysis

To derive the exact solution of the problem (12), we express cos*φ* using the Hamiltonian (13):

$$\begin{array}{c} \text{cos}\varphi =-\frac{{\tilde{I}}_{30}+a_{1}^{4}+4a_{1}^{2}a_{3}^{2}}{4a_{1}^{3}a_{3}}. \end{array}$$ (14)

Let us stress that the inequality

$$\begin{array}{c} |\text{cos}\varphi |\leq 1 \end{array}$$ (15)

must be valid for the problem solution. Further, using the expression (14) and the first equation of the set (12), we write the following equation with respect to the LFW amplitude *a*_1_:

$$\frac{da_{1}}{dz}=\&\tilde{\alpha }a_{1}^{2}a_{3}\sqrt{1-\frac{{\left({\tilde{I}}_{30}+a_{1}^{4}+4a_{1}^{2}a_{3}^{2}\right)}^{2}}{16a_{1}^{6}a_{3}^{2}}}.$$

Multiplying both parts of the equation by *a*_1_ and introducing new notation $p_{1}=a_{1}^{2}$ (corresponding to the LFW intensity), and then substituting the HFW intensity *a*_3_ by using the first invariant: $a_{3}^{2}=1-p_{1}$, we write the differential equation with respect to the intensity of the LFW (*p*_1_):

$$\begin{array}{c} \begin{array}{cc} & \frac{dp_{1}}{dz}=\&\frac{15\gamma^{2}}{2\Delta_{21}k}\sqrt{f(p_{1})},\\ & f\left(p_{1};{\tilde{I}}_{30}\right)=-p_{1}^{4}+1.6p_{1}^{3}+\left(0.24{\tilde{I}}_{30}-0.64\right)p_{1}^{2}-0.32{\tilde{I}}_{30}p_{1}-0.04{\tilde{I}}_{30}^{2}. \end{array} \end{array}$$ (16)

Obviously, the function *f*(*p*_1_) must be non-negative: *f*(*p*_1_)≥0. In fact, this inequality and the inequality (15) are equivalent. To show this, it is necessary to substitute the cos*φ* in the inequality (15). Then, multiplying both parts of the obtained inequality by $4a_{1}^{3}a_{3}$, and substituting *a*_3_ using the first invariant, we obtain the inequality, which both parts are non-negative. After raising both parts to the second power and substituting $a_{1}^{2}$ by *p*_1_, we obtain the inequality *f*(*p*_1_)≥0.

The Eq (16) can be integrated, and its solution crucially depends on roots of the following equation

$$\begin{array}{c} f(p_{1};{\tilde{I}}_{30})=0 \end{array}$$ (17)

as well as on the number of its real roots. For convenience, let us denote these roots as $P_{1_{j}},\;j=1,\;2,\;3,\;4$, and we propose that they satisfy the inequality $P_{1_{1}}\leq P_{1_{2}}\leq P_{1_{3}}\leq P_{1_{4}}$ if all roots are real. In this case, the LFW intensity *P*_1_ changes between roots: $P_{1_{1}}\leq p_{1}\leq P_{1_{2}}$ or $P_{1_{3}}\leq p_{1}\leq P_{1_{4}}$, respectively. In other cases, the intervals of changing LFW amplitude are varied significantly. We will discuss them below at writing the solution of the problem (12).

First of all, we determine a number of the real roots in dependence on the Hamiltonian’s value ${\tilde{I}}_{30}$, which is defined by incident amplitude distributions of the LFW and HFW. Using Sturm theorem for a determination of the real roots number [17], we see that this number is defined by the polynomial:

$$g\left({\tilde{I}}_{30}\right)={\tilde{I}}_{30}^{2}\left({\tilde{I}}_{30}^{2}+3{\tilde{I}}_{30}+1\right)$$

(deriving this polynomial is presented in Appendix C). If the inequality

$$g({\tilde{I}}_{30})>0$$

is satisfied, that takes place for changing the Hamiltonian in the interval

$$-1.5+0.5\sqrt{5}<{\tilde{I}}_{30}<0,$$

then there are four different real roots of the Eq (17). If there is an opposite inequality:

$$g({\tilde{I}}_{30})<0,$$

that corresponds to the Hamiltonian changing in the following interval:

$$-1.5-0.5\sqrt{5}<{\tilde{I}}_{30}<-1.5+0.5\sqrt{5},$$

then there are two different real roots and two complex-value roots.

**Fig 1:**
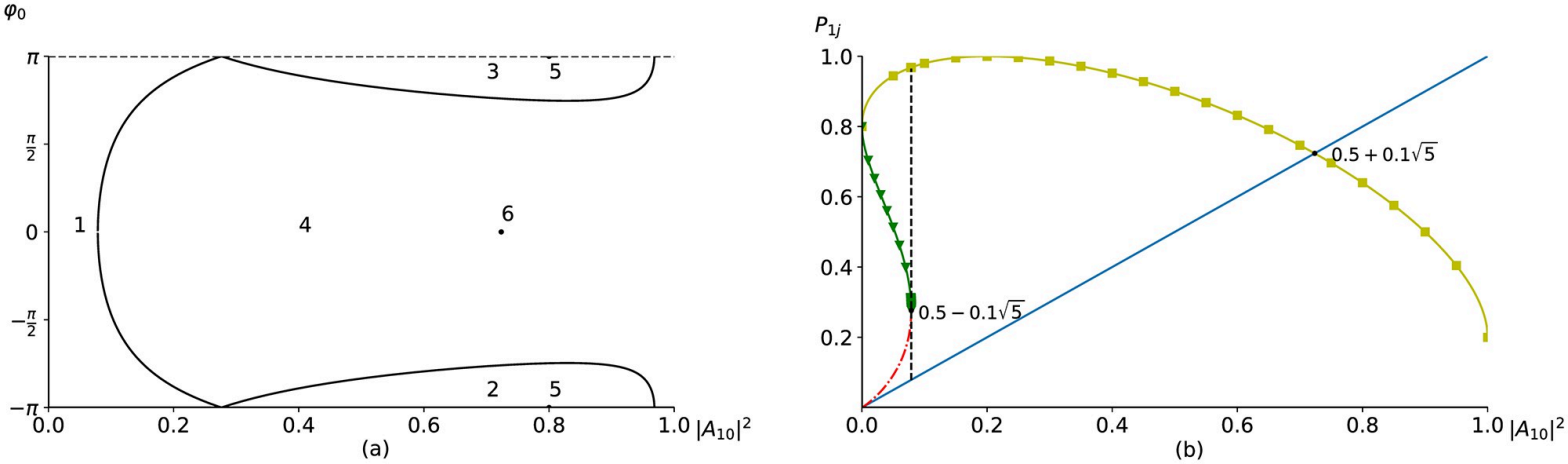
Areas of different number of the problem solution (a): areas 1, 2, 3—four real roots, 4—two real roots and two complex roots, points 5 (0.8, ±*π*)—two multiple real roots, 6 $\left(0.5+0.1\sqrt{5},0\right)$—one multiple real root and two complex roots. Dependence of the roots on the incident LFW intensity at zero-value phase difference *φ*_0_ = 0 (b): $P_{1_{1}}$—blue solid line, $P_{1_{2}}$—red dashed-dotted line, $P_{1_{3}}$—green line with triangles, $P_{1_{4}}$—yellow line with squares.

There are also several special cases if the polynomial $g({\tilde{I}}_{30})$ is equal to zero. So, for

$$\begin{array}{c} {\tilde{I}}_{30}=-1.5-0.5\sqrt{5} \end{array}$$ (18)

there are one two-fold real root $0.5+0.1\sqrt{5}$ and two complex roots. If the Hamiltonian is equal to

$$\begin{array}{c} {\tilde{I}}_{30}=-1.5+0.5\sqrt{5}, \end{array}$$ (19)

then there are one two-fold root $0.5-0.1\sqrt{5}$ and two other different real roots. At ${\tilde{I}}_{30}=0$ there are two two-fold real roots: 0 and 0.8. Because the Hamiltonian is defined by the amplitude and the phase difference of the incident pulses then it is convenient to depict in the plane (|*A*_10_|^2^, *φ*_0_) the areas, possessing different types and number of roots of the (17). Such analysis is shown in Fig 1a.

We see four areas, and one curve, described by the Eq (19) and dividing this plane with respect to |*A*_10_|^2^ and *φ*_0_, and also some points. In the areas 1, 2, 3 there are four real roots of the Eq (17). Thus, two modes of the frequency down-conversion can occur if the incident LFW intensity and the phase difference belonging certain areas: the high-effective mode: $P_{1_{3}}\leq p_{1}\leq P_{1_{4}}$ and the low-effective one $P_{1_{1}}\leq p_{1}\leq P_{1_{2}}$. If the incident LFW intensity is small, then its intensity changes in the low-effective mode: Fig 1b, the LFW intensity changes between blue solid line and red dashed-dotted line. Therefore, the amplification efficiency is low. However, it is possible the high amplification mode of LFW at special choice of its incident intensity. In this case the LFW intensity changes between the green line with triangles and the yellow line with squares.

In the area 4 there are only two real roots, and only one mode of the pulses interaction occurs: the LFW intensity changes between the blue solid line and the yellow line with squares in Fig 1b. The lowest incident intensity |*A*_10_|^2^ = *P*_*min*_ for this mode realization is approximately equal to *P*_*min*_ = 0.07889 at *φ*_0_ = 0. It should be stressed, despite this intensity is computed by using the modified problem, the computer simulation results demonstrate its validity.

Obviously, we want to minimize the incident LFW intensity, at which the high effective mode of its amplification occurs. To determine this minimal value, one needs to solve the equation $\frac{da_{1}}{dz}\left(0\right)=0$. If an intensity of the incident LFW isn’t equal to zero *a*_1_(0)≠0 (necessary condition for beginning its amplification), then this equation requires sin*φ*_0_ = 0 that resulting in *φ*_0_ = 0 or *φ*_0_ = ±*π*. The second derivative of *a*_1_:

$$\begin{array}{c} \frac{d^{2}a_{1}}{dz^{2}}={\tilde{\alpha }}^{2}(18a_{1}^{3}a_{3}^{2}{\text{sin}}^{2}\varphi -9a_{1}^{5}{\text{sin}}^{2}\varphi -3a_{1}^{2}a_{3}\text{cos}\varphi (3(\frac{a_{1}^{3}}{a_{3}}-3a_{1}a_{3})\text{cos}\varphi +3a_{1}^{2}-6a_{3}^{2}+15a_{20}^{2}) \end{array}$$ (20)

in the input section of a medium must be greater than zero if the incident LFW intensity is less than $|A_{10}{|}^{2}<0.5+0.1\sqrt{5}$, which value is achieved at carrying out the equality (18) and corresponds to starting the LFW amplification mode (see Fig 1b). For definiteness, we pay our attention to the case *φ*_0_ = 0 and consider a dependence of the roots $P_{1_{j}}$ on the incident pulse intensity |*A*_10_|^2^. Let us note that the zero-value of the Hamiltonian (Fig 1b) corresponds to two incident LFW intensity |*A*_10_| = 0 and 0.8, which are preserved at the waves interaction: *p*_1_(*z*) = 0 or *p*_1_(*z*) = 0.8 and their changing is absent.

If the incident non-zero LFW intensity is less than |*A*_10_|^2^ < *P*_*min*_, then there are two modes of the LFW amplification. However, in this case, the LFW intensity varies in low-effective mode between the intensities $P_{1_{1}}$ and $P_{1_{2}}$ (Fig 1b). The situation changes dramatically if the incident LFW intensity achieves the value *P*_*min*_. In this case, two roots coincide each other: $P_{1_{2}}=P_{1_{3}}=0.5-0.1\sqrt{5}$, and any small increasing of its intensity results in the explosive growth of the LFW intensity. Therefore, a rigorous mode of the LFW amplification occurs. If the incident LFW intensity |*A*_10_|^2^ exceeds *P*_*min*_, then two roots of the Eq (17) become complex ones. Therefore, there is only one mode of waves interaction and the LFW intensity *p*_1_ changes between the intensity values $P_{1_{1}}$ and $P_{1_{4}}>0.8$. Thus, the amplification of the LFW intensity becomes very effective. Let’s note that the root $P_{1_{4}}$ achieves a value equal unity at the incident IFW intensity |*A*_20_|^2^ = 0.2, and then it decreases and coincides with the $P_{1_{1}}$ root at the incident LFW intensity $|A_{10}{|}^{2}=0.5+0.1\sqrt{5}$.

If the parameters of the incident waves intensities correspond to the points 5 and 6 in Fig 1b, then the LFW intensity remains unchanged and equals 0.8 or $0.5+0.1\sqrt{5}$, respectively. Another remark refers to the possibility of achieving high-effective amplification of the LFW by introducing phase difference *φ* of the interacting waves at certain sections of a medium if even the incident LFW intensity is small. But such discussion is far from aim of this paper.

In the end of this section, we give the formulas describing LFW intensity evolution and also some computer simulation results confirming analytical results. Many of them contain the elliptical functions: an elliptical cosine *cn*(*z*, *k*) and elliptical sine *sn*(*z*, *k*), but the elliptical function *dn*(*z*, *k*) does not appear in those formulas.

**Case:** *P*_12_, *P*_13_—**complex roots** ($-1.5-0.5\sqrt{5}<{\tilde{I}}_{30}<-1.5+0.5\sqrt{5}$).

Let us denote *r* = *Re*(*P*_12_), *s* = *Im*(*P*_12_) and introduce new notations:

$$\begin{array}{cc} & c=\sqrt{{\left(P_{14}-r\right)}^{2}+s^{2}},\;d=\sqrt{{\left(P_{11}-r\right)}^{2}+s^{2}},\\ & \kappa =\sqrt{cd},k=\frac{\kappa^{2}+\left(r-P_{11}\right)\left(P_{14}-r\right)-s^{2}}{2\kappa^{2}}. \end{array}$$

Then the LFW intensity evolution is described by formula

$$\begin{array}{c} p_{1}\left(z\right)=\frac{\left(cP_{11}-dP_{14}\right)cn\left(7.5\tilde{\alpha }\kappa z,k\right)+\left(cP_{11}+dP_{14}\right)}{\left(c-d\right)cn\left(7.5\tilde{\alpha }\kappa z,k\right)+\left(c+d\right)}. \end{array}$$ (21)

**Case of four real roots:** 0 < *P*_11_ < *P*_12_ < *P*_13_ < *P*_14_ < 1 ($-1.5+0.5\sqrt{5}<{\tilde{I}}_{30}<0$).

If the incident LFW intensity is enough high, then its intensity *p*_1_ belongs to an interval (*P*_13_, *P*_14_) (high-effective mode occurs) at its propagation in a medium and the intensity evolution is described by the following formula:

$$\begin{array}{c} \begin{array}{cc} & p_{1}\left(z\right)=\frac{\left(P_{14}-P_{13}\right)P_{12}sn^{2}\left(3.75\tilde{\alpha }\sqrt{\left(P_{14}-P_{12}\right)\left(P_{13}-P_{11}\right)}z,k\right)-\left(P_{14}-P_{12}\right)P_{13}}{\left(P_{14}-P_{13}\right)sn^{2}\left(3.75\tilde{\alpha }\sqrt{\left(P_{14}-P_{12}\right)\left(P_{13}-P_{11}\right)}z,k\right)-\left(P_{14}-P_{12}\right)},\\ & k=\sqrt{\frac{\left(P_{14}-P_{13}\right)\left(P_{12}-P_{11}\right)}{\left(P_{14}-P_{12}\right)\left(P_{13}-P_{11}\right)}}. \end{array} \end{array}$$ (22)

In opposite case, the LFW intensity *p*_1_(*z*) amplification varies in low-effective mode and its intensity changes between values *P*_11_ and *P*_12_ in accordance with the formula:

$$\begin{array}{c} \begin{array}{cc} & p_{1}\left(z\right)=\frac{\left(P_{12}-P_{11}\right)P_{14}sn^{2}\left(3.75\tilde{\alpha }\sqrt{\left(P_{14}-P_{12}\right)\left(P_{13}-P_{11}\right)}z,k\right)+\left(P_{14}-P_{12}\right)P_{11}}{\left(P_{12}-P_{11}\right)sn^{2}\left(3.75\tilde{\alpha }\sqrt{\left(P_{14}-P_{12}\right)\left(P_{13}-P_{11}\right)}z,k\right)+\left(P_{14}-P_{12}\right)},\\ & k=\sqrt{\frac{\left(P_{14}-P_{13}\right)\left(P_{12}-P_{11}\right)}{\left(P_{14}-P_{12}\right)\left(P_{13}-P_{11}\right)}}. \end{array} \end{array}$$ (23)

**Special case of multiple roots** 0 < *P*_11_ < *P*_12_ = *P*_13_ < *P*_14_ < 1 (${\tilde{I}}_{30}=-1.5+0.5\sqrt{5}$).

In this case, the LFW intensity *p*_1_ evolution on *z*−coordinate occurs in the following way:

$$\begin{array}{c} \begin{array}{cc} & z\left(p_{1}\right)=\frac{1}{7.5\tilde{\alpha }\sqrt{\left(P_{14}-P_{13}\right)\left(P_{13}-P_{11}\right)}}\text{ln}\frac{{\left(\sqrt{\left(P_{13}-P_{11}\right)\left(P_{14}-p_{1}\right)}+\sqrt{\left(P_{14}-P_{13}\right)\left(p_{1}-P_{11}\right)}\right)}^{2}}{|p_{1}-P_{13}|\left(P_{14}-P_{11}\right)}. \end{array} \end{array}$$ (24)

It should be stressed that under writing of the formula (24) we neglect the terms possessing order *O*((Δ_21_ *k*)^−2^) (and lower). However, if the waves interaction mode corresponds to a case of the multiple roots then an influence of small terms increases many times. As a result, the solution of the original problem may be periodical one with large period while the solution of a set of the modified equation is aperiodic.

For instance, if the pump pulse intensity *p*_1_(*z*) starts to change from its value *P*_11_ or *P*_14_, then the LFW intensity reaches intensity *P*_13_ at z-coordinate tending to infinity in accordance with the formula (24). In contrast, the computer simulation results demonstrate periodic or quasi-periodic mode of the LFW intensity evolution. However, at tending |*A*_10_|^2^ to *P*_*min*_, the period of oscillations for LFW intensity grows (though remains bounded). As we can see from analysis made above, the LFW intensity oscillation period is defined by the Hamiltonian, whose value, in turn, is defined by the intensities of the incident waves and the phase difference between them.

#### 4.1.2 Computer simulation results

Below we present computer simulation results, which confirms the derived formulas. The conversion efficiency is defined as ratio between the LFW intensity and the sum of intensities for the incident LFW and HFW:

$$\eta \left(z\right)=\frac{|A_{1}{\left(z\right)|}^{2}}{|A_{1}{\left(0\right)|}^{2}+{\left|A_{3}\left(0\right)\right|}^{2}}.$$

We apply this formula even if the incident IFW intensity is not non-zero.

**Fig 2:**
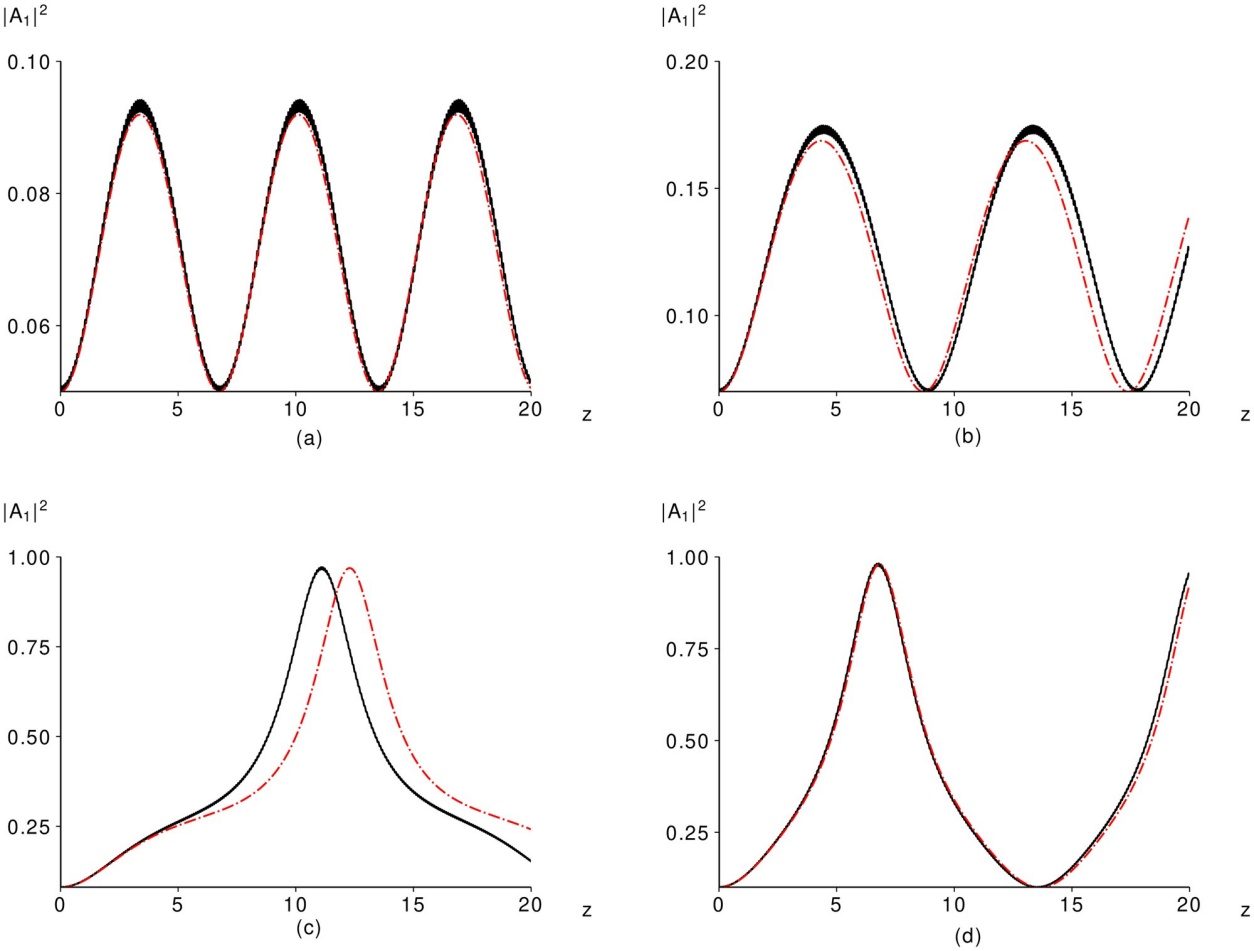
Computer simulation results obtained on the base of both the original problem (black solid lines) and the modified problem (red dashed-dotted lines) computed for the parameters: *γ* = 4, Δ_21_ *k* = 80, |*A*_20_|^2^ = 0, *φ*_0_ = 0 and |*A*_10_|^2^ = 0.05 (a), 0.07 (b), 0.08 (c), 0.1 (d).

As example, Fig 2a–2d illustrate the low-efficient amplification of LFW and the high-efficient one that occurring in dependence on the incident LFW intensity. The pulses interaction distance equals 5 dimensionless units corresponding to the crystal length of 2 cm in our notations.

If the incident LFW intensity is equal to |*A*_10_|^2^ = 0.05, then the maximal LFW intensity achieves relatively low value (0.12). A period of the intensity oscillations equals 6 dimensionless units. The first maximum of the LFW intensity achieves in the section *z* = 3. We see that the solution of the modified problem perfectly approximate the original problem solution.

At the incident LFW intensity |*A*_10_|^2^ = 0.07 (Fig 2b), which is a little lower than *P*_*min*_, the maximal LFW intensity is quite low (approximately 0.18). However, this intensity is achieved in the bigger section (*z* = 4.5) of a medium in comparison with the previous case (Fig 2a).

If the LFW incident intensity |*A*_10_|^2^ is greater than the critical one *P*_*min*_, then the maximal LFW intensity growths sufficiently in accordance with the results of the theoretical analysis. Such case (|*A*_10_|^2^ = 0.08) is depicted in Fig 2c. The maximal LFW intensity achieves practically unity (0.972) at the pulses propagation distance *z* = 11. It should be emphasized that a difference between the intensities’ evolution in Fig 2c is caused by a proximity of the incident LFW intensity to the critical one (*P*_*min*_). In this case, it is necessary take into account the next terms in a series on Δ_21_ *k*. Partly, it can be caused by presence of the IFW which is not took into account in the modified equations.

In contrast to previous two Figs., the LFW intensity evolution is described by complicated function and the inflexion point occurring approximately at the intensity being equal to $|A_{1}{\left(z\right)|}^{2}=0.5-0.1\sqrt{5}$ exists. At this intensity value, the switching between the low-efficiency amplification mode and high those occurs. Due to reasons, mentioned above, the multi-scale method approximates worse (Fig 2c) than in other cases: the maximal intensity computed using the modified problem is achieved in the section *z* = 12.2 and equals 0.968. So, from the physical point of view, the solution of the original problem gives even preferable results (lower distance and bigger intensity) than the solution of the modified problem. Nevertheless, the simplified equations predict the maximal LFW intensity and the modes switching.

If the LFW incident intensity is increased until |*A*_10_|^2^ = 0.1 (Fig 2d), then the simplified equations approximate again perfectly the pulses interaction because its value sufficiently far from the critical intensity *P*_*min*_. As we can see in Fig 2d, there is not a saturation of the frequency conversion efficiency with increasing propagation distance. The saturation of the frequency conversion occurs, for example, for the second harmonic generation analyzed in the framework of the long pulse duration and plane wave approximation. This process was described in the well-known paper [18]. The saturation may appear also if the frequency conversion is analyzed in the framework of the pump non-depletion approximation. We do not use this approximation. We derived an explicit solution of the modified equations in the framework of the long pulse duration and plane wave approximation. As follows from (12), the phase difference depends on ratio of the intensities of the interacting waves. Therefore, changing of the phase difference can lead to inverse energy transfer: from the LFW to HFW. This is a reason of the LFW intensity evolution depicted in Fig 2d.

### 4.2 General case: *a*_20_ ≠ 0

#### 4.2.1 Analysis

After previous analysis of the particular case, we briefly describe the general case. By expressing cos*φ* through the Hamiltonian:

$$\text{cos}\varphi =\frac{2a_{20}^{2}\left(4a_{1}^{2}-a_{3}^{2}\right)-{\tilde{I}}_{30}-a_{1}^{4}-4a_{1}^{2}a_{3}^{2}}{4a_{1}^{2}a_{3}^{2}}$$

and then providing similar algebra, one can obtain the differential equation with respect to intensity *p*_1_ of the LFW:

$$\begin{array}{c} \begin{array}{cc} & \frac{dp_{1}}{dz}=\&\frac{15\gamma^{2}}{2\Delta_{21}k}\sqrt{f(p_{1})},\\ & f\left(p_{1}\right)=-p_{1}^{4}+\left(0.64+0.48B\right)p_{1}^{3}+\left(0.24A-0.16B^{2}\right)p_{1}^{2}-0.16ABp_{1}-0.04A^{2},\\ & A=\tilde{I_{30}}+2a_{20}^{2},\;B=2-5a_{20}^{2}. \end{array} \end{array}$$ (25)

For simplicity, we introduce new parameters *A* and *B*. To demonstrate an influence of the incident IFW intensity on the LFW amplification, we compute the zeros of the polynomial *f*(*p*_1_) = 0 (see (25)) occurring for the incident LFW intensity belonging to 0 ≤ |*A*_10_|^2^ ≤ 0.1 in dependence of |*A*_10_|^2^ and |*A*_20_|^2^. We choose these LFW intensity values because the critical intensity *P*_*min*_ for the mode switching for LFW amplification is less than 0.1 and we want to follow changing the critical intensity *P*_*min*_ with increasing the incident IFW intensity, which belongs to an interval 0 ≤ |*A*_20_|^2^ ≤ 0.5.

**Fig 3:**
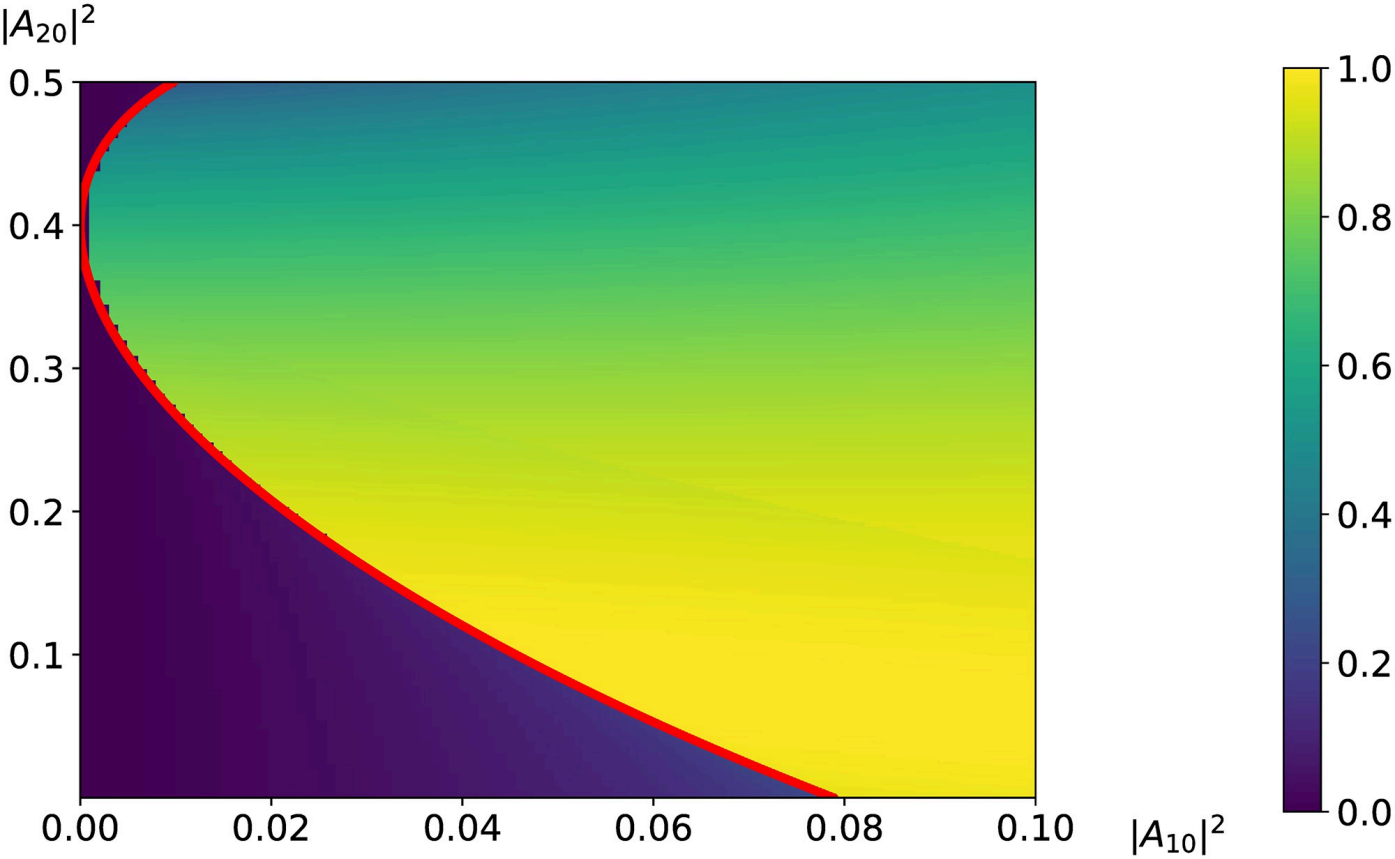
Dependence of the LFW maximal intensity on the incident LFW intensity and the incident IFW intensity. The boundary between high-effective mode and low-effective mode is depicted with red solid line.

In Fig 3 the dependence of the maximal LFW intensity on incident intensities of LFW and IFW is depicted on the plane (|*A*_10_|^2^, |*A*_20_|^2^) at the chosen phase difference *φ*_0_ of the incident pulses being equal to 0 or *π* to get a positive value of the second order derivative from the LFW amplitude: $\frac{\partial^{2}a_{1}}{\partial z^{2}}>0$.

Let us remind that to obtain the maximal LFW intensity amplification at zero-value incident IFW intensity, the phase difference *φ*_0_ must be chosen equal zero if the LFW incident intensity belongs to interval $0<|A_{10}{|}^{2}<0.5+0.1\sqrt{5}$. However, at non-zero-value incident IFW intensity, in accordance with the differential Eq (20) (the second order derivative must be positive), the LFW incident intensity interval decreases with growing IFW incident intensity |*A*_20_|^2^ and the phase difference *φ*_0_ must be equal to *π* if the IFW intensity satisfies the inequality: |*A*_20_|^2^ ≥ 0.4.

It should be stressed that at the incident IFW intensity |*A*_20_|^2^ = 0.4 it is possible to achieve large LFW intensity for very low (even zero) its incident one. In this case, the LFW intensity is governed by the formula, which does not meet in the previous section. Let us discuss in detail this important case. The roots of the polynomial (25) are computed from the equation:

$$-p_{1}^{3}\left(25p_{1}-16\right)=0.$$

There are a three-fold root equal zero and one non-zero-value root 0.64. It means that the LFW generation does not start from its zero-value intensity. However, if the incident intensity of LFW is non-zero then its intensity in a medium changes in accordance with the formula:

$$\begin{array}{c} \begin{array}{cc} & p_{1}\left(z\right)=\frac{16}{{\left(24\tilde{\alpha }z-z_{1}\right)}^{2}+25},\\ & z_{1}=\sqrt{\frac{16-25|A_{10}{|}^{2}}{|A_{10}{|}^{2}}}. \end{array} \end{array}$$ (26)

Thus, the LFW intensity *p*_1_(*z*), changing as reverse quadratic function, has one maximum and the required distance for its achievement tends to infinity if |*A*_10_|→0. We note that this conclusion can not be applied directly to the original problem due to the same reasons as the formula (24) discussed above. Nevertheless, the formulas (26) shows that with decreasing LFW incident intensity, the required crystal length grows.

Let us analyze Fig 3, in which the boundary between low-effective amplification mode and high-effective amplification mode is depicted by red line and this line is governed by the solution of the equation:

$$\begin{array}{cc} & 140625A^{5}-52500A^{4}B^{2}+641250A^{4}B-275625A^{4}-1850A^{3}B^{4}-178200A^{3}B^{3}\\ & +1177350A^{3}B^{2}-530550A^{3}B-677475A^{3}-276A^{2}B^{6}-4308A^{2}B^{5}-165060A^{2}B^{4}\\ & +897210A^{2}B^{3}-174690A^{2}B^{2}-1088208A^{2}B-381024A^{2}+529AB^{8}-3864AB^{7}\\ & +1530AB^{6}-36786AB^{5}+242325AB^{4}+24912AB^{3}-425952AB^{2}-311040AB\\ & -62208A+1058B^{9}-8211B^{8}+14830B^{7}+11520B^{6}-22080B^{5}-20736B^{4}-4608B^{3}=0, \end{array}$$

where the parameters *A* and *B* were introduced in the formula (25). It is easy to see that the values $A=-1.5+0.5\sqrt{5},\;B=2$, which corresponds to the incident intensities |*A*_10_|^2^ = *P*_*min*_ and |*A*_20_|^2^ = 0, satisfy this equation. If the IFW incident intensity |*A*_20_|^2^ increases then the intensity *P*_*min*_, corresponding to switching between modes of the LFW amplification, decreases. On the other hand, the maximal amplification of the LFW decreases relatively slow. If the IFW incident intensity is equal to |*A*_20_|^2^ = 0.4, then the critical intensity for switching of LFW mode amplification is equal to zero: *P*_*min*_ = 0. Further, if the IFW incident intensity is greater than 0.4 dimensionless units (|*A*_20_|^2^ > 0.4), then the critical intensity *P*_*min*_ increases. It is not suitable for our aim, therefore, |*A*_20_|^2^ should be chosen less or equal to 0.4. In fact, all the solutions from the previous section are also valid for the case under consideration with substitution zeros of the polynomial from (16) to the zeros of the polynomial from (25).

#### 4.2.2 Computer simulation results

**Fig 4:**
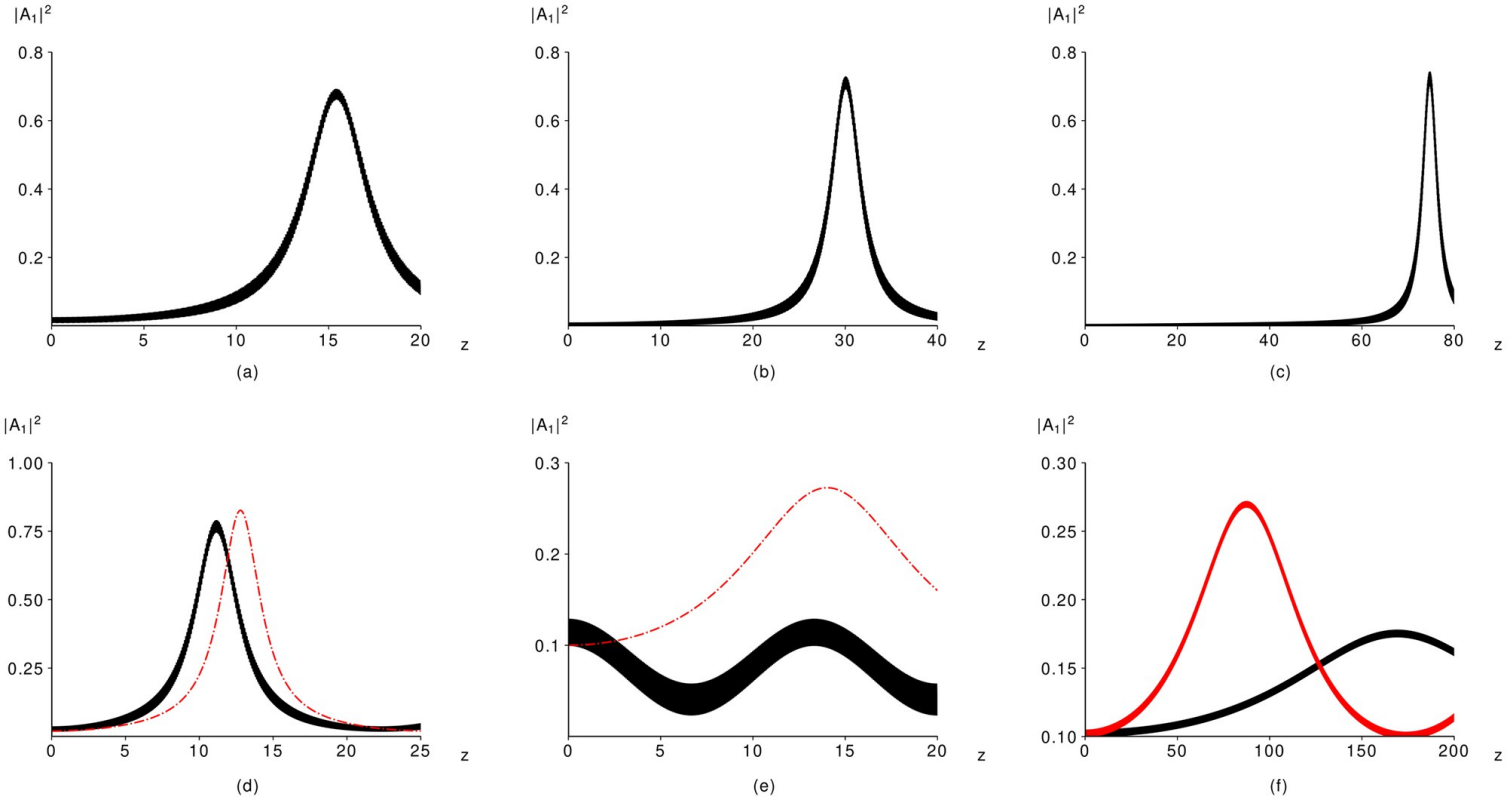
Computer simulation results of the original problem (black solid lines) and the modified problem (red dashed-dotted lines) at *γ* = 4, Δ_21_ *k* = 80 (a- e), 500 (f), *φ*_0_ = 0 and (|*A*_10_|^2^, |*A*_20_|^2^ = (0.01, 0.4) (a), (0.001, 0.4) (b), (0, 0.4) (c), (0.02, 0.3) (d), (0.1, 0.5) (e,f).

First of all, we discuss the computer simulation results provided at enough large incident intensity of the IFW |*A*_20_|^2^ = 0.4, at which the strong LFW amplification can be achieved even at its very low incident intensity (Fig 4). In this case, the crystal length, required for achieving the high efficiency of the frequency conversion, crucially depends on the LFW incident intensity and this length is much greater in comparison with the case of the zero-value IFW incident intensity. Fig 4a–4c, depicted for |*A*_10_|^2^ equal 0.01;0.001;0.0, respectively, confirms the theoretical results. In this case, practically 70% of the HFW energy converts to the LFW energy. However, the required distance is a little larger than in the previous paragraph.

One may notice that the solid line, which depict the LFW intensity evolution, looks thick in Fig 4. The reason is the fast oscillations of the LFW intensity, caused by the non-zero IFW incident intensity. They also exist at |*A*_20_| = 0, but in the case under consideration they are much stronger.

With decreasing incident LFW intensity (Fig 4b), the required crystal length increases two times (*z* = 31). However, the maximal intensity becomes a little bit large. In practice, one can even choose *A*_10_ = 0 (Fig 4c), but we see that the required crystal length becomes too much long (*z* = 70), and maybe it is inconvenient for practice.

We must notice that generally speaking, the multi-scale method approximate worse the original solution if the incident IFW intensity is non-zero: |*A*_20_|^2^ ≠ 0. As a rule, the difference in maximal intensity or the required distance of its achievement predicted by using this approximation and computed on the base of the original problem may appear, as it is shown in Fig 4d. However, there are some cases, when two solutions do not coincide at all. Such case is presented in Fig 4e. We see that while the LFW intensity computed using the modified problem increases, the solution of the original problem decreases. Nevertheless, there are some ways to improve the multi-scale approximation in this case.

First of all, one may use additional terms corresponding to other scales (*O*(Δ_21_ *k*^−2^) and lower). To prove this, we essentially increase the phase mismatching Δ_21_ *k* until value 500. As follows from Fig 4f, both intensities grow without large oscillations in contrast to the previous case (Fig 4e). However, maximal intensities and coordinates of their achievement differ significantly. Thus, the solution developed using multi-scale method must contain additional scales at certain values of the problem parameters.

## 5 Frequency down-conversion under accounting for the dispersion of non-linear coupling coefficient

Let us briefly discuss the frequency down-conversion under accounting for different values of second-order susceptibilities because the components of the *χ*^(2)^ tensor depend on are different as well-known. Our aim is a demonstration of effective applying multi-scale method in this case also. In the long pulse duration approximation, the original problem (1) can be re-written as [19]:

$$\begin{array}{c} \begin{array}{cc} & \frac{dA_{1}}{dz}+i(\gamma_{1}A_{1}^{*}A_{2}e^{-i\Delta_{21}kz}+\gamma_{2}A_{2}^{*}A_{3}e^{i\Delta_{21}kz})=0,\\ & \frac{dA_{2}}{dz}+i(\gamma_{1}A_{1}^{2}e^{i\Delta_{21}kz}+2\gamma_{2}A_{1}^{*}A_{3}e^{i\Delta_{21}kz})=0,\\ & \frac{dA_{3}}{dz}+3i\gamma_{2}A_{1}A_{2}e^{-i\Delta_{21}kz}=0,\; \end{array} \end{array}$$ (27)

For brevity, below, we will call *γ*_1_ as SHG coefficient and *γ*_2_ as DFG coefficient.

After applying the multi-scale method, we obtain the following set of modified equations:

$$\begin{array}{c} \begin{array}{cc} & \frac{dU}{dz}-i(\frac{\gamma_{1}^{2}}{\Delta_{21}k}{\left|U\right|}^{2}U+3\frac{\gamma_{1}\gamma_{2}}{\Delta_{21}k}U^{*2}W+2\frac{\gamma_{2}^{2}}{\Delta_{21}k}{U|W|}^{2})=0,\\ & \frac{dW}{dz}-3i(\frac{\gamma_{1}\gamma_{2}}{\Delta_{21}k}U^{3}+2\frac{\gamma_{2}^{2}}{\Delta_{21}k}{\left|U\right|}^{2}W)=0, \end{array} \end{array}$$ (28)

Energy’s invariant *I*_*UW*_ has the same view as above, but the Hamiltonian is written as:

$$I_{3}=-3(4\frac{\gamma_{1}\gamma_{2}}{\Delta_{21}k}Re\left(U^{3}W^{*}\right)+\frac{\gamma_{1}^{2}}{\Delta_{21}k}{\left|U\right|}^{4}+4\frac{\gamma_{2}^{2}}{\Delta_{21}k}{\left|U\right|}^{2}{\left|W\right|}^{2})=const.$$

Then, we use the representation (11) to obtain the following set of equations:

$$\begin{array}{c} \begin{array}{cc} & \frac{da_{1}}{dz}=-3\frac{\gamma_{1}\gamma_{2}}{\Delta_{21}k}a_{1}^{2}a_{3}\text{sin}\varphi ,\\ & \frac{da_{3}}{dz}=3\frac{\gamma_{1}\gamma_{2}}{\Delta_{21}k}a_{1}^{3}\text{sin}\varphi ,\\ & \frac{d\varphi }{dz}-(3\frac{\gamma_{1}\gamma_{2}}{\Delta_{21}k}(\frac{a_{1}^{3}}{a_{3}}-3a_{1}a_{3})\text{cos}\varphi +(6\frac{\gamma_{2}^{2}}{\Delta_{21}k}-3\frac{\gamma_{1}^{2}}{\Delta_{21}k})a_{1}^{2}-6\frac{\gamma_{2}^{2}}{\Delta_{21}k}a_{3}^{2})=0,\\ & a_{1}\left(0\right)=|A_{10}|,\;a_{3}\left(0\right)=\left|A_{30}\right|,\;\varphi \left(0\right)=\varphi_{0}. \end{array} \end{array}$$ (29)

In new variables, the Hamiltonian takes the form:

$$I_{3}=3(-4\frac{\gamma_{1}\gamma_{2}}{\Delta_{21}k}a_{1}^{3}a_{3}\text{cos}\varphi -\frac{\gamma_{1}^{2}}{\Delta_{21}k}a_{1}^{4}-4\frac{\gamma_{2}\gamma_{2}}{\Delta_{21}k}a_{1}^{2}a_{3}^{2}),$$

which is divided on $3\gamma_{2}^{2}/\Delta_{21}k$ to write the modified Hamiltonian:

$${\tilde{I}}_{3}=-4qa_{1}^{3}a_{3}\text{cos}\varphi -q^{2}a_{1}^{4}-4a_{1}^{2}a_{3}^{2}={\tilde{I}}_{30}.$$

Here, the parameter q is ratio between SHG and DFG coefficients: *q* = *γ*_1_/*γ*_2_.

Using this invariant and energy’s invariant *I*_1*UW*_, we derive the equation with respect to LFW intensity *p*_1_:

$$\begin{array}{cc} & \frac{dp_{1}}{dz}=\&\frac{3\gamma_{1}\gamma_{2}}{4\Delta_{21}k}\sqrt{f(p_{1})},\\ & f\left(p_{1};{\tilde{I}}_{30},\;q\right)=-{\left(q^{2}+4\right)}^{2}p_{1}^{4}+\left(8q^{2}+32\right)p_{1}^{3}+\left(16-2\left(q^{2}-4\right){\tilde{I}}_{30}-0.64\right)p_{1}^{2}-8{\tilde{I}}_{30}p_{1}-{\tilde{I}}_{30}^{2}. \end{array}$$

**Fig 5:**
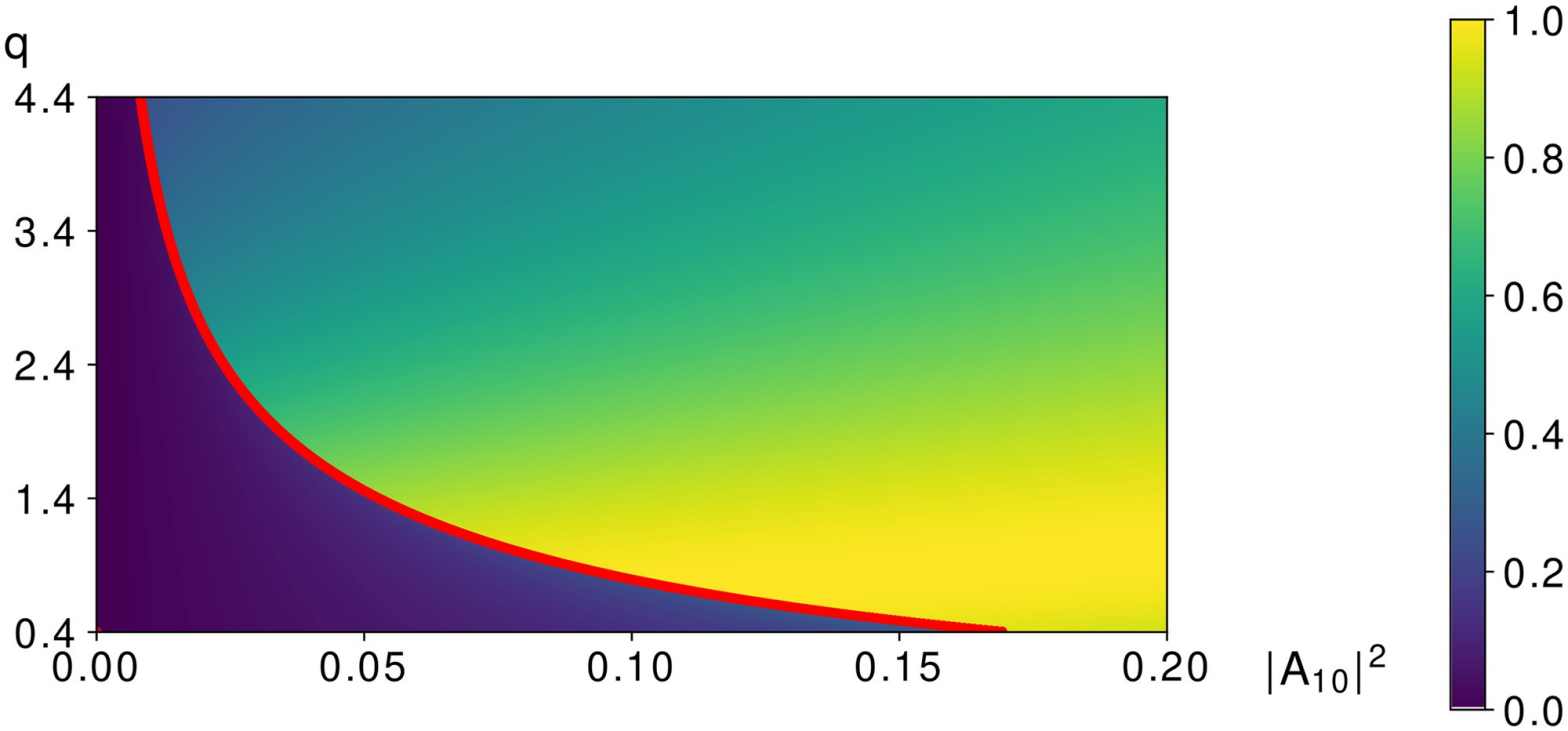
Dependence of the LFW maximal intensity on the incident LFW intensity and the ratio between the SHG coefficient and DFG coefficient. The boundary between high-effective mode and low-effective mode is depicted by red solid line

The analysis of this equation shows that the critical value of the LFW intensity *P*_*min*_ decreases if the SHG coefficient prevails over DFG coefficient as illustrate Fig 5. The border between the low-effective mode and high-effective mode is shown in this Fig. by the following equation:

$$I_{30}^{2}+I_{30}q^{2}+2I_{30}+1=0.$$

**Fig 6:**
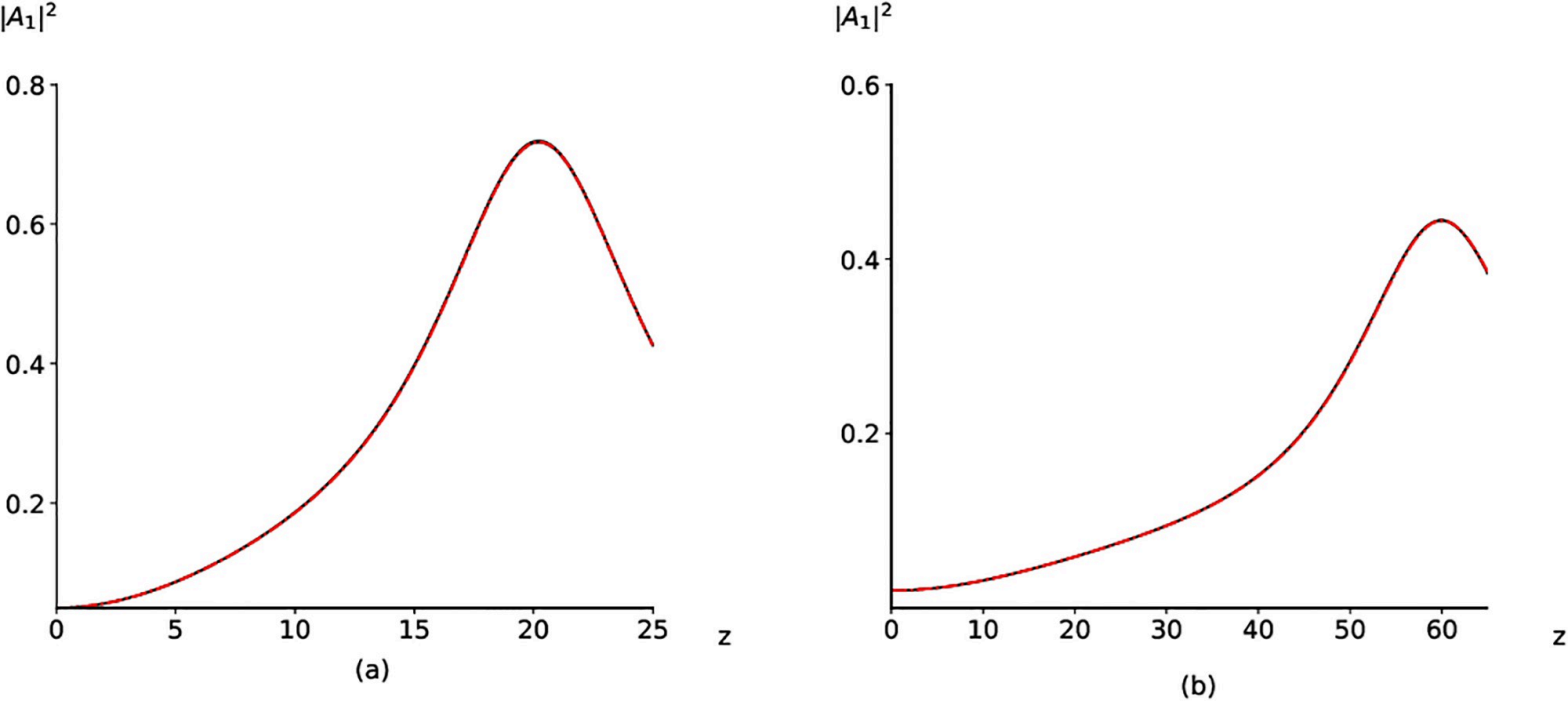
Computer simulation results obtained by using the original problem (black solid lines) and the modified problem (red dashed-dotted lines) computed for the parameters *γ*_1_ = 4, Δ_21_ *k* = 80, |*A*_20_|^2^ = 0, *φ*_0_ = 0 and (*γ*_2_, |*A*_10_|^2^) = (2, 0.05) (a), $\left(0.02,\;\frac{4}{3}\right)$ (b).

Without detail theoretical analysis, let us present only the computer simulation results depicted in Fig 6). Comparison of Fig 6a with Fig 2a demonstrates that the maximal intensity of the LFW increases dramatically if the parameter *q* is changed from unity until two. In the last case, the LFW maximal intensity achieves a value 0.73, which is six times greater than at *q* = 1.

We emphasize that it is possible even to decrease the incident LFW intensity if q equals 3 (|*A*_20_|^2^ = 0.02, Fig 6b). However, in this case, some disadvantages appear. Firstly, the conversion efficiency (45%) is smaller than in previous cases. Secondly, the required distance for its achieving grows until *z* = 60.

Thus, the dispersion of the quadratic susceptibility may improve a process of the frequency down-conversion.

## 6 Conclusion

We showed the possibility of high efficient frequency down-conversion (3*ω*, 2*ω* → *ω*) based on cascading process of the three-waves interaction in a medium with quadratic non-linear response. The phase matching occurs between the HFW and LFW. The IFW propagates under large phase mismatching between this wave and other waves.

Using multi-scale method, we derived a set of the modified equations, which qualitatively and quantitatively describe the LFW amplification. Based on these equations, we analyzed various modes of the waves interaction and showed that the interaction possesses a property of bistability: there are high- and low-efficiency modes of the LFW amplification.

We considered two cases: the incident IFW is present or absent. In both cases, the high intensity of LFW can be achieved if certain conditions are valid. If the IFW incident intensity equals zero, then the incident LFW intensity must be greater than crucial value to achieve a large amplification of this wave. In opposite case, the crucial incident intensity of the LFW may be decreased and at certain incident intensity of the IFW, the LFW amplification occurs even for the incident LFW zero-value intensity. However, the incident IFW large intensity causes the fast oscillations of the LFW intensity.

Based on provided analysis, one can propose two stages amplification scheme. In the first crystal, there is a generation of the LFW (wave with the half-frequency) under the phase matching between IFW and LFW. It occurs until the LFW intensity achieves a value being equal or greater than *P*_*min*_. Then the LFW and HFW fall into the second crystal under the condition of both the phase matching between HFW and LFW and big phase mismatching between HFW and IFW. In this crystal, the high-efficient amplification of the LFW occurs.

Because we did not take into account the group-velocity mismatching and the group-velocity dispersion, our results can be used for the pulses with long duration (such as picosecond, nanosecond, microsecond or even CW). Using of the pulses with short duration must be accompanied satisfying well-known conditions on the crystal length, non-linear length, and lengths characterizing group velocities mismatching and the second order dispersion of the pulse.

Our computer simulation results showed that the HFW energy can be practically fully converted to the LFW energy under certain condition for the incident LFW intensity which satisfies the equality: |*A*_10_|^2^ > *P*_*min*_, or that is about 70% if the incident LFW intensity is equal to zero and the incident IFW intensity is equal to certain value. Both these conversion efficiency values are greater than the corresponding values obtained previously (34%, [8]). There are also other possibilities for high-efficient frequency down-conversion if the incident intensities of both waves are non-zero.

Briefly we discuss the possible set of the parameters for a realization of results, obtained in this paper. For example, at using AgGaS_2_ crystal, and for a sum of the incident intensities 0.12 GW/cm^2^, the required crystal length equals about 3 cm if the incident LFW intensity is remarkable (i.e bigger than *P*_*min*_ equaling 9 MW/cm^2^, approximately) or 8–18 cm if this intensity is close to zero. We stress that the crystal length decreases in accordance with the linear law at growing incident intensities of the pulses. If the incident intensity of the pump pulse increases four times then the required distances decrease to 0.7 cm and 4–9 cm, respectively.

Thus, our approach may be useful for obtaining high-efficient generation of the IR radiation and even THz radiation.

## Appendix A: Derivation of modified equations

Here we derive the equation set (9) which describes an appearance of the induced cubic nonlinear response at big phase mismatching between IFW and LFW: |Δ_21_ *k*|> > 1. In this case, the process of wave interaction possesses various space scales: in particular, a small scale, defined by big phase mismatching |Δ_21_ *k*|, and a long space scale defined by the dispersion lengths of the interacting pulses. Let us introduce a small parameter $\mu =\frac{1}{\Delta_{21}k}$ (for simplicity, we suppose that the phase mismatching has a positive sign) and introduce various scales along *z* coordinate: small scale equal to the inverse phase mismatching length: $\xi =\frac{z}{\mu }$, and big longitudinal scales *z*_*l*_ = *μ*^*l*^ *z*, *l* = 0, 1, 2…. Therefore, the complex amplitudes are expanded in a power series of *μ*:

$$\begin{array}{c} \begin{array}{cc} & A_{1}=U+\mu U_{1}+\mu^{2}U_{2}+...,\\ & A_{2}=V+\mu V_{1}+\mu^{2}V_{2}+...,\\ & A_{3}=W+\mu W_{1}+\mu^{2}W_{2}+.... \end{array} \end{array}$$ (30)

Obviously, the functions in (30) depend on all the variables (*t*, *ξ*, *z*_*l*_|*l* ≥ 0).

Using the chain rule, we write differential operators in terms of new variables:

$$\begin{array}{c} \begin{array}{cc} L_{j} & =\frac{\partial }{\partial z}+\nu_{j1}\frac{\partial }{\partial t}+iD_{j}\frac{\partial^{2}}{\partial t^{2}}=\frac{\partial \xi }{\partial z}\frac{\partial }{\partial \xi }+\sum_{l=0}^{\infty }\frac{\partial z_{l}}{\partial z}\frac{\partial }{\partial z_{l}}+iD_{j}\frac{\partial^{2}}{\partial t^{2}}=\frac{1}{\mu }\frac{\partial }{\partial \xi }\\ & +\sum_{l=0}^{\infty }\mu^{l}\frac{\partial }{\partial z_{l}}+iD_{j}\frac{\partial^{2}}{\partial t^{2}}=\frac{1}{\mu }\frac{\partial }{\partial \xi }+L_{j}^{0}+\mu \frac{\partial }{\partial z_{1}}+\mu^{2}\frac{\partial }{\partial z_{2}}+...,j=1,2,3. \end{array} \end{array}$$ (31)

Here, operator *L*_*j*_ is defined as

$$L_{j}^{\left(0\right)}=\frac{\partial }{\partial z_{0}}+\nu_{j1}\frac{\partial }{\partial t}+iD_{j}\frac{\partial^{2}}{\partial t^{2}}.$$

Then, we substitute the expansion (30) into the equation set (1), and write all terms with an order, which is greater than *μ*^2^:

$$\begin{array}{c} \begin{array}{cc} & \frac{1}{\mu }\frac{\partial U}{\partial \xi }+L_{1}^{\left(0\right)}U+\mu \frac{\partial U}{\partial z_{1}}+\frac{\partial U_{1}}{\partial \xi }+\mu L_{1}^{\left(0\right)}U_{1}+\mu \frac{\partial U_{2}}{\partial \xi }+\\ + & i\gamma (U^{*}Ve^{-i\xi }+V^{*}We^{i\xi }+\mu \left(\left(U^{*}V_{1}+U_{1}^{*}V\right)e^{-i\xi }+\left(V^{*}W_{1}+V_{1}^{*}W\right)e^{i\xi }\right))+O\left(\mu^{2}\right)=0,\\ & \frac{1}{\mu }\frac{\partial V}{\partial \xi }+L_{2}^{\left(0\right)}V+\mu \frac{\partial V}{\partial z_{1}}+\frac{\partial V_{1}}{\partial \xi }+\mu L_{1}^{\left(0\right)}V_{1}+\mu \frac{\partial V_{2}}{\partial \xi }+\\ + & i\gamma (U^{2}e^{i\xi }+2U^{*}We^{i\xi }+\mu \left(2UU_{1}e^{i\xi }+\left(U^{*}W_{1}+U_{1}^{*}W\right)e^{i\xi }\right))+O\left(\mu^{2}\right)=0,\\ & \frac{1}{\mu }\frac{\partial W}{\partial \xi }+L_{3}^{\left(0\right)}W+\mu \frac{\partial W}{\partial z_{1}}+\frac{\partial W_{1}}{\partial \xi }+\mu L_{1}^{\left(0\right)}W_{1}+\mu \frac{\partial W_{2}}{\partial \xi }+\\ + & 3i\gamma (UVe^{-i\xi }+\mu \left(UV_{1}+U_{1}V\right)e^{-i\xi })+O\left(\mu^{2}\right)=0. \end{array} \end{array}$$ (32)

Grouping the terms with respect to power of *μ* we obtain the equations:

$$\frac{\partial U}{\partial \xi }=\frac{\partial V}{\partial \xi }=\frac{\partial W}{\partial \xi }=0,$$

corresponding to $\frac{1}{\mu }$ power of the expansion. Consequently, the functions *U*, *V* and *W* do not depend on fast changing coordinate *ξ*. Therefore, these functions do not change at the small scale.

For the next order *O*(1) of power *μ*, we obtain the following set of equations:

$$\begin{array}{c} \begin{array}{cc} & L_{1}^{\left(0\right)}U+\frac{\partial U_{1}}{\partial \xi }+i\gamma \left(U^{*}Ve^{-i\xi }+V^{*}We^{i\xi }\right)=0,\\ & L_{2}^{\left(0\right)}V+\frac{\partial V_{1}}{\partial \xi }+i\gamma \left(U^{2}e^{i\xi }+2U^{*}We^{i\xi }\right)=0,\\ & L_{3}^{\left(0\right)}W+\frac{\partial W_{1}}{\partial \xi }+3i\gamma UVe^{-i\xi }=0. \end{array} \end{array}$$ (33)

So, since the first terms in these equations do not depend on *ξ*, meanwhile other terms do depend on this variable, we can separate equations into two parts. The first of them is written as

$$\begin{array}{c} L_{1}^{\left(0\right)}U=L_{2}^{\left(0\right)}V=L_{3}^{\left(0\right)}W=0. \end{array}$$ (34)

The functions *U*_1_, *V*_1_, *W*_1_ can be found from the second one by integrating (33) with respect to *ξ*:

$$\begin{array}{c} \begin{array}{cc} & U_{1}=\gamma \left(U^{*}Ve^{-i\xi }-V^{*}We^{i\xi }\right)+u_{1}\left(t,z_{0},\;z_{1}...\right),\\ & V_{1}=\gamma \left(-U^{2}e^{i\xi }-2U^{*}We^{i\xi }\right)+v_{1}\left(t,z_{0},\;z_{1}...\right),\\ & W_{1}=3\gamma UVe^{-i\xi }+w_{1}\left(t,z_{0},\;z_{1}...\right). \end{array} \end{array}$$ (35)

Here *u*_1_, *v*_1_, *w*_1_ are the function of integration: they do not depend on *ξ*. The equations, which they are governed by, are derived further.

At the order *O*(*μ*), the equations are the following:

$$\begin{array}{cc} & \frac{\partial U_{2}}{\partial \xi }+L_{1}^{\left(0\right)}U_{1}+\frac{\partial U}{\partial z_{1}}+i\gamma \left(\left(U^{*}V_{1}+U_{1}^{*}V\right)e^{-i\xi }+\left(V^{*}W_{1}+V_{1}^{*}W\right)e^{i\xi }\right)=0,\\ & \frac{\partial V_{2}}{\partial \xi }+L_{2}^{\left(0\right)}V_{1}+\frac{\partial V}{\partial z_{1}}+i\gamma \left(2UU_{1}e^{i\xi }+\left(U^{*}W_{1}+U_{1}^{*}W\right)e^{i\xi }\right)=0,\\ & \frac{\partial W_{2}}{\partial \xi }+L_{3}^{\left(0\right)}W_{1}+\frac{\partial W}{\partial z_{1}}+3i\gamma \left(UV_{1}+U_{1}V\right)e^{-i\xi }=0. \end{array}$$

Using the representation (35) this set transforms into the form:

$$\begin{array}{cc} & \frac{\partial U_{2}}{\partial \xi }+\gamma \left(L_{1}^{\left(0\right)}\left(U^{*}V\right)e^{-i\xi }-L_{1}^{\left(0\right)}\left(V^{*}W\right)e^{i\xi }\right)+i\gamma^{2}(U^{*}v_{1}e^{-i\xi }-V^{2}W^{*}e^{-2i\xi }+u_{1}^{*}Ve^{-i\xi }+V^{*}w_{1}e^{i\xi }\\ + & v_{1}^{*}We^{i\xi })=-(\frac{\partial U}{\partial z_{1}}+i\gamma^{2}{\left(-|U\right|}^{2}U-3U^{*2}W+{4U|V|}^{2}-{2U|W|}^{2}))-L_{1}^{\left(0\right)}u_{1},\\ & \frac{\partial V_{2}}{\partial \xi }-\gamma \left(L_{2}^{\left(0\right)}\left(U^{2}\right)e^{i\xi }+2L_{2}^{\left(0\right)}\left(U^{*}W\right)e^{i\xi }\right)+i\gamma^{2}(-2UV^{*}We^{2i\xi }+2Uu_{1}e^{i\xi }+2UV^{*}We^{i\xi }\\ + & 2u_{1}^{*}We^{i\xi }+2U^{*}we^{i\xi })=-(\frac{\partial V}{\partial z_{1}}+2i\gamma^{2}\left(4|U\right|-{\left|W\right|}^{2})V)-L_{2}^{\left(0\right)}v_{1},\\ & \frac{\partial W_{2}}{\partial \xi }+3\gamma L_{3}^{\left(0\right)}\left(UV\right)e^{-i\xi }+3i\gamma^{2}\left(Uv_{1}e^{-i\xi }+U^{*}V^{2}*e^{-2i\xi }+u_{1}V_{1}e^{-i\xi }\right)=\\ - & (\frac{\partial W}{\partial z_{1}}-3i\gamma^{2}(U^{3}+{2|U|}^{2}W+{\left|V\right|}^{2}W))-L_{1}^{\left(0\right)}w_{1}. \end{array}$$

As before, we can state that the right-hand sides of the equations are equal to zero because they do not depend on *ξ* in contrast to the left-hand sides of the equations. Thus, we write the equations

$$\begin{array}{cc} & \frac{\partial U}{\partial z_{1}}+i\gamma^{2}{\left(-|U\right|}^{2}U-3U^{*2}W+{4U|V|}^{2}-{2U|W|}^{2})=-L_{1}^{\left(0\right)}u_{1},\\ & \frac{\partial V}{\partial z_{1}}+2i\gamma^{2}{\left(4|U\right|}^{2}-{\left|W\right|}^{2})V=L_{2}^{\left(0\right)}v_{1},\\ & \frac{\partial W}{\partial z_{1}}-3i\gamma^{2}(U^{3}+{2|U|}^{2}W+{\left|V\right|}^{2}W=L_{3}^{\left(0\right)}w_{1}. \end{array}$$

Here, we separate terms, which contain *u*_1_, *v*_1_, *w*_1_. Since in the representation (30) they belong to order *O*(*μ*), meanwhile, *U*, *V*, *W* belong to order *O*(1), then we can once again separate the obtained equation into two parts:

$$\begin{array}{c} \begin{array}{cc} & \frac{\partial U}{\partial z_{1}}+i\gamma^{2}{\left(-|U\right|}^{2}U-3U^{*2}W+{4U|V|}^{2}-{2U|W|}^{2})=0,\\ & \frac{\partial V}{\partial z_{1}}+2i\gamma^{2}{\left(4|U\right|}^{2}-{\left|W\right|}^{2})V=0,\\ & \frac{\partial W}{\partial z_{1}}-3i\gamma^{2}(U^{3}+{2|U|}^{2}W+{\left|V\right|}^{2}W)=0. \end{array} \end{array}$$ (36)

Consequently, the equations

$$\begin{array}{c} \begin{array}{cc} & L_{1}^{\left(0\right)}u_{1}=0,\\ & L_{2}^{\left(0\right)}v_{1}=0,\\ & L_{3}^{\left(0\right)}w_{1}=0 \end{array} \end{array}$$ (37)

are valid.

After returning to original variables ($\xi =\Delta_{21}kz,\;z_{0}=z,\;z_{1}=z/\Delta_{21}k,\;\frac{\partial }{\partial z}=\Delta_{21}k\frac{\partial }{\partial \xi }+\frac{\partial }{\partial z_{0}}+\frac{1}{\Delta_{21}k}\frac{\partial }{\partial z_{1}}+O\left({\left(\Delta_{21}k\right)}^{-2}\right)$), we obtain the sets of Eq (5) and (6). In turn, the expansion series (30) transforms into the form (4).

## Appendix B: Alternative approach to derivation of modified equations

The derivation of the modified equations, presented in the Appendix A, is quite complicated, or, maybe even hard for understanding their major features. In order to illustrate the essence of cascading processes, we give another (more simple) derivation of the modified equations, for example, in the case |*A*_20_|^2^ = 0. This approach leads to the same results as at using multi-scale method if the IFW is not of interest for us.

So, let us represent the complex amplitude *A*_2_ as a series:

$$A_{2}=\left(A_{2}^{\left(0\right)}+A_{2}^{\left(1\right)}+A_{2}^{\left(2\right)}+...\right)e^{i\Delta_{21}kz},$$

where each of the terms corresponds to 1/(Δ_21_ *k*)^−*m*^. Substituting this series into the second equation of the system (1) (we still suppose that Δ_31_ *k* = 0), we obtain the following equation in the first order of the approximation:

$$\frac{\partial A_{2}^{\left(0\right)}}{\partial z}+\nu_{21}\frac{\partial A_{2}^{\left(0\right)}}{\partial t}+iD_{2}\frac{\partial A_{2}^{\left(0\right)}}{\partial^{2}t^{2}}+i\Delta_{21}kA_{2}^{\left(0\right)}+i\gamma \left(A_{1}^{2}+2A_{1}^{*}A_{3}\right)=0,$$

which can be re-written as:

$$A_{2}^{\left(0\right)}+\frac{\gamma }{\Delta_{21}k}\left(A_{1}^{2}+2A_{1}^{*}A_{3}\right)-\frac{i}{\Delta_{21}k}\frac{\partial^{2}A_{2}^{\left(0\right)}}{\partial z}+\frac{\nu_{21}}{\Delta_{21}k}\frac{\partial A_{2}^{\left(0\right)}}{\partial t}+i\frac{D_{2}}{\Delta_{21}k}\frac{\partial A_{2}^{\left(0\right)}}{\partial t^{2}}=0.$$

One can see that the last two terms contain both complex amplitude $A_{2}^{\left(0\right)}$ and (Δ_21_ *k*)^−1^. Therefore, they are much smaller than other terms. Therefore, they can be neglected. As follows, we obtain the following relation:

$$\begin{array}{c} A_{2}^{\left(0\right)}=-\frac{\gamma }{\Delta_{21}k}\left(A_{1}^{2}+2A_{1}^{*}A_{3}\right). \end{array}$$ (38)

Here we can see the phase grating corresponding to the IFW. Its weak generation is caused by the large phase mismatching Δ_21_ *k*. Nevertheless, it plays essential role because other two waves are scattering on this phase grating.

Then substituting representation (38) into the first equation and the third Eq (1), we write the equations:

$$\begin{array}{cc} & \frac{\partial A_{1}}{\partial z}+iD_{1}\frac{\partial^{2}A_{1}}{\partial t^{2}}+i\frac{\gamma^{2}}{\Delta_{21}k}\left(-\right|A_{1}{|}^{2}U-3A_{1}^{*2}A_{3}-2A_{1}{\left|A_{3}\right|}^{2}=0,\\ & \frac{\partial A_{3}}{\partial z}+\nu_{31}\frac{\partial A_{3}}{\partial t}+iD_{3}\frac{\partial^{2}A_{3}}{\partial t^{2}}-3i\frac{\gamma^{2}}{\Delta_{21}k}(A_{1}^{3}+2|A_{1}{|}^{2}A_{3})+i\Delta_{31}kA_{3}=0, \end{array}$$

which coincides fully with (5), if we suppose *V* ≡ 0 (this, in turn, follows from |*A*_20_|^2^ = 0).

## Appendix C: Computation of the real roots number of the equation *f*(*p*_1_) = 0

Here we illustrate a computation of the real zeros’ number of the polynomial (17). For this aim we use Sturm theorem. So, first of all, we write so-called Sturm sequence, which is written in the following view:

$$\begin{array}{cc} & f_{0}\left(p_{1}\right)=-f\left(p_{1}\right)=p_{1}^{4}-1.6p_{1}^{3}-\left(0.24{\tilde{I}}_{30}-0.64\right)p_{1}^{2}+0.32{\tilde{I}}_{30}p_{1}+0.04{\tilde{I}}_{30}^{2},\\ & f_{1}\left(p_{1}\right)=-f^{'}\left(p_{1}\right)=4p_{1}^{3}-4.8p_{1}^{2}-\left(0.24{\tilde{I}}_{30}-1.28\right)p_{1}+0.32{\tilde{I}}_{30},\\ & f_{2}\left(p_{1}\right)=\left(0.12{\tilde{I}}_{30}+0.16\right)p_{1}^{2}+\left(-0.192{\tilde{I}}_{30}-0.64\right)p_{1}-0.04{\tilde{I}}_{30}^{2}-0.032{\tilde{I}}_{30},\\ & f_{3}\left(p_{1}\right)=\frac{-192{\tilde{I}}_{30}^{3}-1216{\tilde{I}}_{30}^{2}-512{\tilde{I}}_{30}}{225{\tilde{I}}_{30}^{2}+600{\tilde{I}}_{30}+400}p_{1}+\frac{-192{\tilde{I}}_{30}^{3}-128{\tilde{I}}_{30}^{2}}{225{\tilde{I}}_{30}^{2}+600{\tilde{I}}_{30}+400},\\ & f_{4}\left(p_{1}\right)=\frac{9{\tilde{I}}_{30}^{6}+51{\tilde{I}}_{30}^{5}+97{\tilde{I}}_{30}^{4}+72{\tilde{I}}_{30}^{3}+16{\tilde{I}}_{30}^{2}}{225{\tilde{I}}_{30}^{4}+2850{\tilde{I}}_{30}^{3}+10225{\tilde{I}}_{30}^{2}+7600{\tilde{I}}_{30}+1600} \end{array}$$

in the case under consideration. This sequence is obtained on the base of the Euclid’s algorithm for polynomials division: functions *f*_*j*_, *j* = 2, 3, 4 are division residues of *f*_*j*−2_ on *f*_*j*−1_, and then multiplied on -1. As the Sturm theorem states, the number of real roots of *f*(*p*_1_) = 0 in the interval [a,b] equals the difference

n(a)-n(b),

if *f*(*a*)≠0 and *f*(*b*)≠0, where n(x) is the number of sign changes in the Sturm sequence *f*_*j*_ at the point *x* (zeros do not count).

We have to answer two questions. Firstly, how many real roots of the equation *f*(*p*_1_) = 0 exist in the interval [0, 1] because the LFW intensity is bounded between 0 and 1 due to normalization introduced by us. Secondly, how many real roots of the equation *f*(*p*_1_) = 0 exist because this defines the formula describing the intensities evolution. Based on the Sturm theorem conditions, first we must consider two special cases: *f*(0) = 0 and *f*(1) = 0. In the first case, ${\tilde{I}}_{30}=0$ and there are two two-fold roots 0 and 0.8. In the second case, ${\tilde{I}}_{30}=-1$ and there are two complex roots and two real roots 0.2 and 1.

Now we analyze the case *f*(0)≠0, *f*(1)≠0 corresponding to ${\tilde{I}}_{30}\neq 0,\;{\tilde{I}}_{30}\neq 1$. Therefore, the Sturm theorem can be applied. In Table 1 we present values of the functions *f*_*j*_(*p*_1_), *j* = 0..5 for the arguments 0 and 1, and show their coefficients at highest degree of the polynomial (−1)^*j*^ *f*_*j*_(*p*_1_), *j* = 0..4 because these coefficients define the signs of the functions *f*_*j*_ at tending *p*_1_ to ±∞. For definiteness, we denote them as *sgn*(*f*_*j*_(±∞)), *j* = 0..4, respectively, and *n*(±∞) means a short notation of lim*n*(*x*) at tending of *x* to ±∞.

**Table 1:** Values of functions *f*_*j*_ at certain points.

	*sgn*(*f*_*j*_(−∞))	*f*_*j*_(0)	*f*_*j*_(1)	*sgn*(*f*_*j*_(+ ∞))
*f*_0_	1	0.04${\tilde{I}}_{30}^{2}$	$0.04{\tilde{I}}_{30}^{2}+0.08{\tilde{I}}_{30}+0.04$	1
*f*_1_	-4	$0.32{\tilde{I}}_{30}$	$-0.16{\tilde{I}}_{30}+0.48$	4
*f*_2_	$0.12{\tilde{I}}_{30}+0.16$	$-0.04{\tilde{I}}_{30}^{2}-0.032{\tilde{I}}_{30}$	$-0.04{\tilde{I}}_{30}^{2}-0.104{\tilde{I}}_{30}+0.032$	$0.12{\tilde{I}}_{30}+0.16$
*f*_3_	$\frac{192{\tilde{I}}_{30}^{3}+1216{\tilde{I}}_{30}^{2}+512{\tilde{I}}_{30}}{225{\tilde{I}}_{30}^{2}+600{\tilde{I}}_{30}+400}$	$\frac{-192{\tilde{I}}_{30}^{3}-128{\tilde{I}}_{30}^{2}}{225{\tilde{I}}_{30}^{2}+600{\tilde{I}}_{30}+400}$	$\frac{-384{\tilde{I}}_{30}^{3}-1344{\tilde{I}}_{30}^{2}-512{\tilde{I}}_{30}}{225{\tilde{I}}_{30}^{2}+600{\tilde{I}}_{30}+400}$	$\frac{-192{\tilde{I}}_{30}^{3}-1216{\tilde{I}}_{30}^{2}-512{\tilde{I}}_{30}}{225{\tilde{I}}_{30}^{2}+600{\tilde{I}}_{30}+400}$
*f*_4_	$\frac{9{\tilde{I}}_{30}^{6}+51{\tilde{I}}_{30}^{5}+97{\tilde{I}}_{30}^{4}+72{\tilde{I}}_{30}^{3}+16{\tilde{I}}_{30}^{2}}{225{\tilde{I}}_{30}^{4}+2850{\tilde{I}}_{30}^{3}+10225{\tilde{I}}_{30}^{2}+7600{\tilde{I}}_{30}+1600}$			

Thus, we are able to compute the function *n*(*x*) at chosen argument. Obviously, these values depend on the Hamiltonian ${\tilde{I}}_{30}$ and they are shown in Table 2.

**Table 2:** Number of sign changes *n*(*x*) of the Sturm sequence for different values of the Hamiltonian ${\tilde{I}}_{30}$.

${\tilde{I}}_{30}$	*n*(−∞)	*n*(0)	*n*(1)	*n*(+ ∞)
${\tilde{I}}_{30}=-1.5-0.5\sqrt{5}$	2	2	1	1
$-1.5-0.5\sqrt{5}<{\tilde{I}}_{30}<-1$	3	3	1	1
$-1<{\tilde{I}}_{30}<-1.5+0.5\sqrt{5}$	3	3	1	1
${\tilde{I}}_{30}=-1.5+0.5\sqrt{5}$	3	3	0	0
$-1.5+0.5\sqrt{5}<{\tilde{I}}_{30}<0$	4	4	0	0

Let us remind that the Sturm theorem can not be implemented at ${\tilde{I}}_{30}=0$ and ${\tilde{I}}_{30}=-1$. Therefore, these values are absent in Table 2 and they are discussed above separately. We notice that there are other values of the third invariant ${\tilde{I}}_{30}$, at which *f*_*j*_, *j* = 1..5 can change their signs for *p*_1_ = 0, 1 or ±∞. However, there are only a few values of the Hamiltonian among which the function *n*(*x*) changes its values. These values are presented in Table in Table 2.

All real roots of the equation *f*(*p*_1_) = 0 belong to the interval [0, 1] because *n*(−∞)−*n*(0) = 0 and *n*(+ ∞)−*n*(1) = 0 for all ${\tilde{I}}_{30}$. A number of the real roots belonging to the interval [0, 1] for different ${\tilde{I}}_{30}$ is already described in the main part of the paper.
